# CTC-Race: Single-Cell Motility Assay of Circulating Tumor Cells from Metastatic Lung Cancer Patients

**DOI:** 10.1021/acsnano.3c09450

**Published:** 2024-03-11

**Authors:** Yang Liu, Wujun Zhao, Jamie Hodgson, Mary Egan, Christen N. Cooper Pope, Glenda Hicks, Petros G. Nikolinakos, Leidong Mao

**Affiliations:** † School of Chemical, Materials and Biomedical Engineering, College of Engineering, The University of Georgia, Athens, Georgia 30602, United States; ‡ FCS Technology, LLC, Athens, Georgia 30602, United States; § University Cancer and Blood Center, LLC, Athens, Georgia 30607, United States; ∥ School of Electrical and Computer Engineering, College of Engineering, The University of Georgia, Athens, Georgia 30602, United States

**Keywords:** circulating tumor cells, cell motility, cell migration, single-cell analysis, cancer metastasis

## Abstract

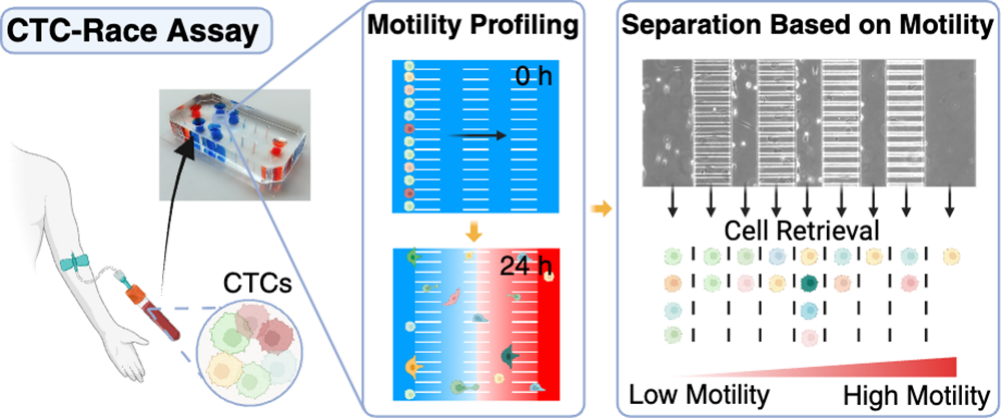

Distinctive subpopulations of circulating tumor cells (CTCs) with increased motility are considered to possess enhanced tumor-initiating potential and contribute to metastasis. Single-cell analysis of the migratory CTCs may increase our understanding of the metastatic process, yet most studies are limited by technical challenges associated with the isolation and characterization of these cells due to their extreme scarcity and heterogeneity. We report a microfluidic method based on CTCs’ chemotactic motility, termed as CTC-Race assay, that can analyze migrating CTCs from metastatic non-small-cell lung cancer (NSCLC) patients with advanced tumor stages and enable concurrent biophysical and biochemical characterization of them with single-cell resolution. Analyses of motile CTCs in the CTC-Race assay, in synergy with other single cell characterization techniques, could provide insights into cancer metastasis.

Individual and cluster of circulating tumor cells (CTCs) hold promise as a research tool for metastatic cancer, which is responsible for over 90% of cancer-related deaths.^1−3^ The multifaceted role of CTCs in metastasis is under intensive investigations.^3−8^ Next-generation sequencing has revealed discordance in the genetic and transcriptional profiles between CTCs and their primary tumors,^9−16^ suggesting that distinctive tumor cell subpopulations with enhanced tumor-initiating potentials may exist and contribute to metastasis.^1−3,11,17^ Such analysis faces challenges from the extreme scarcity of tumor cells in circulation and a lack of isolation and characterization technologies with increased sensitivity to accommodate cellular heterogeneity.^4,6^ CTCs are highly heterogeneous in their biological and biophysical characteristics with multiple phenotypes coexisting and evolving dynamically over the course of metastasis.^3,11^ It is believed that only a small fraction of cancer cells in blood circulation is capable of seeding new tumors.^2,3,18^ These tumor-initiating cells are conferred with a repertoire of distinct biological traits that enable them to disassociate from the primary tumor, invade the surrounding stroma, intravasate and extravasate the circulatory system, and invade a distant organ.^1−3,18^ Notably, increased cell motility is central among these traits and represents integral components of the metastatic process.^19−22^ As such, single-cell analysis of motile CTCs with the potential to start new tumors could provide valuable insights into cancer metastasis. However, with the scarcity and heterogeneity of CTCs in patient samples limiting single-cell motility analysis, current studies have resorted to cultured cancer cells rather than patient-derived cells.^23−27^

Here we developed the CTC-Race assay, a microfluidic platform with multiplexed single-cell racetracks and sustained chemokine gradients, for the biophysical and biochemical analyses of motile CTCs from cancer patients’ blood samples. The CTC-Race assay enables concurrent separation of motile cells from a heterogeneous population of tumor and blood cells relying on cells’ chemotactic motility and quantification of the biological and biophysical characteristics of the motile CTCs with single-cell resolution. Motile CTCs can be extracted from the device for potential molecular and genetic characterization. We validated the utility of the CTC-Race assay with multiple cancer cell lines and CTCs derived from four lung cancer patients.

## Results and Discussion

### Overview of CTC-Race Assay

**Figure 1:**
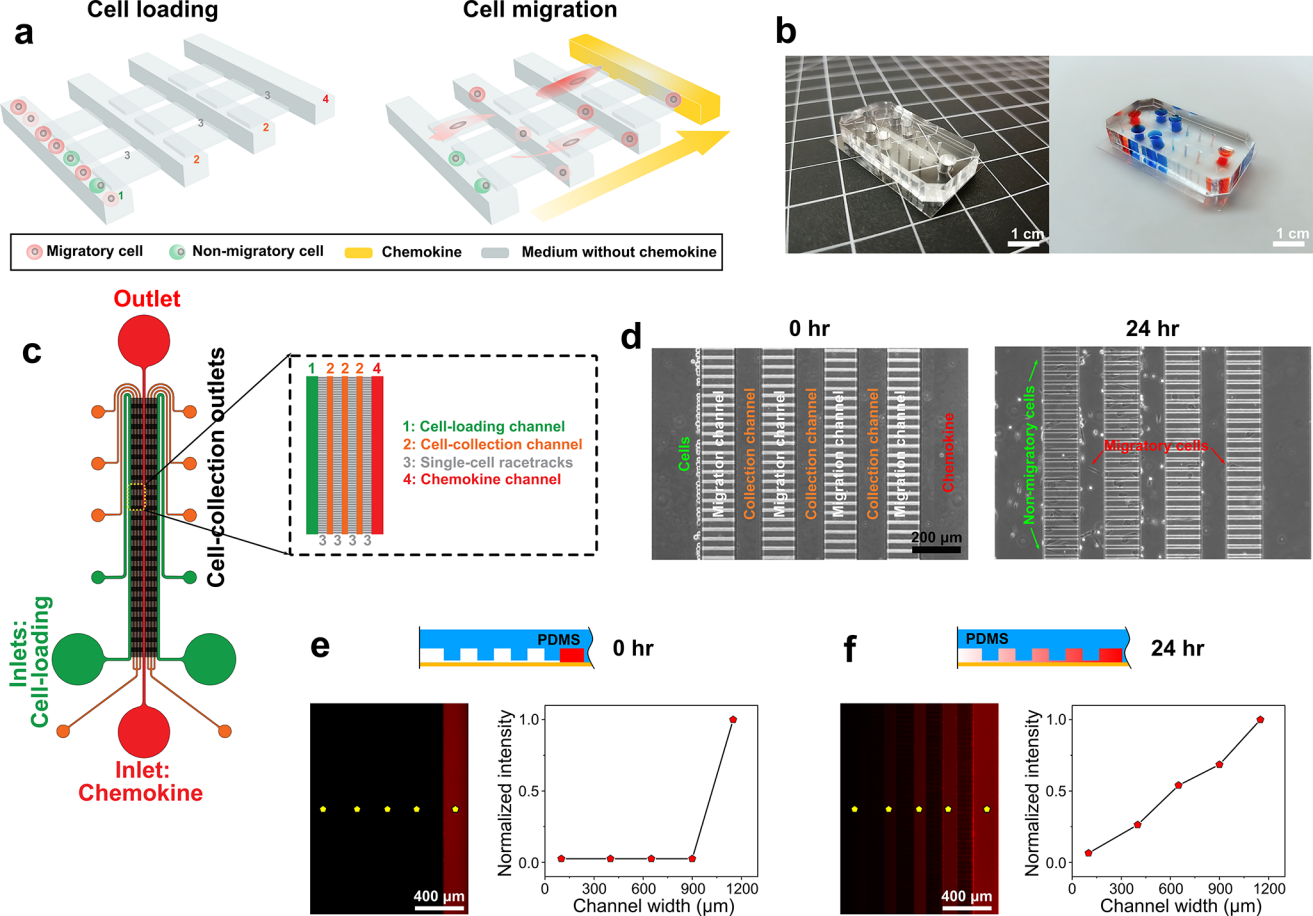
Overview of the CTC-Race assay and its device. (a) Schematic of CTC-Race assay. The microfluidic device comprises a cell-loading channel (1), multiple single-cell racetrack segments (3), multiple cell-collection channels (2), and a chemokine channel (4). The height of single-cell racetracks is designed to be smaller than the diameter of cancer cells so that cancer cells are trapped at the entrance of single-cell racetracks at the start of the assay. Migratory cells (red) squeeze into the tracks and migrate toward the region with a high concentration of chemoattractants, while the nonmigratory cells (green) are trapped in the cell-loading channel during the assay. Cell-collection channels are inserted into the single-cell racetracks perpendicularly to enable cell retrieval. (b) Photos of a PDMS-based CTC-Race device (right, blue and orange colors indicate the channel geometry). (c) Top view schematic of the CTC-Race device. Cells are injected via the cell-loading inlets, and chemokines are continuously perfused through the chemokine channel. The device has a symmetric design with each side having a cell-loading channel (width of 200 μm, height of 50 μm), three cell-collection channels (width of 100 μm, height of 50 μm), and four segments of single-cell racetracks (each segment’s length, 150 μm, width of 30 μm, height of 5 μm). There are 2500 single-cell racetracks on each side and 5000 racetracks in total in one device. Both sides share a chemokine channel (width of 200 μm, height of 50 μm). (d) Phase-contrast images of the migration pattern of MDA-MB-231 breast cancer cells in the CTC-Race device. Chemokine channel is loaded with serum (fetal bovine serum, FBS) as a chemoattractant. (e) Top, side view of rhodamine B (red) concentration at *t* = 0 h. Bottom left, a fluorescent image of the device at *t* = 0 h of rhodamine B perfusion with a flow rate of 0.1 μL/min. Bottom right, normalized fluorescence intensity across the device at the selected positions in the device (yellow dots on the fluorescent image) at *t* = 0 h. (f) Top, side view of rhodamine B (red) at *t* = 24 h. Bottom left, a fluorescent image of the device at *t* = 24 h. Bottom right, normalized fluorescence intensity across the device at *t* = 24 h.

CTC-Race assay, a microfluidic device with 5000 single-cell race tracks and sustained chemokines’ gradient, is developed in order to analyze motile CTCs from patient samples (Figure 1a). The assay is designed to be integrated with existing CTC enrichment tools to obtain adequate and functional tumor cells from blood samples from which the subpopulation of motile cells is isolated and characterized. We integrated the CTC-Race assay with a high-throughput label-free CTC isolation tool, inertial-ferrohydrodynamic cell separation,^28^ that selected CTCs based on cell size and produced an adequate number of CTCs for the race assay. In constructing the microfluidic device for the CTC-Race assay (Figure 1b), we considered the following design principles. First, the collagen-coated (Figure S1) single-cell racetracks in the polydimethylsiloxane (PDMS)-on-glass device are fabricated to recapitulate the confined space through which tumor cells infiltrate organs.^19^ The device consists of multiplexed parallel single-cell racetracks connected by perpendicular cell-loading channels to maximize the number of cells that enter the racetracks to migrate (Figure 1c). The racetracks mimic dimensions of tunnel-like tracks tumor cells encounter in the extracellular matrix (ECM) of the tumor stroma, with each track having a cross-section of 30 μm (width) by 5 μm (height) and a total length of 1200 μm. The single-cell racetracks are periodically interrupted by perpendicular cell-collection channels to enable the retrieval of migrating cells throughout the assay duration (Figure 1d). Second, chemokine gradients are used to guide CTC’s movement in the racetracks because CTCs are most efficient when the cell is involved in directed migration.^29^ A spatial gradient of serum and/or growth factors is maintained via continuous perfusions to enable chemotactic migration of cancer cells or CTCs (Figures 1e,f). We tested the stability of chemokine gradients within the device and found that they were established within minutes of the perfusion and remained constant for up to 24 h (Figure S2). With these designs, the CTC-Race assay enables quantitative measurements at a single-cell resolution of cell migration/speed, cell aspect ratio, and biomarker expressions.

### Functional Analyses of Cultured Cancer Cells in the CTC-Race Assay

**Figure 2:**
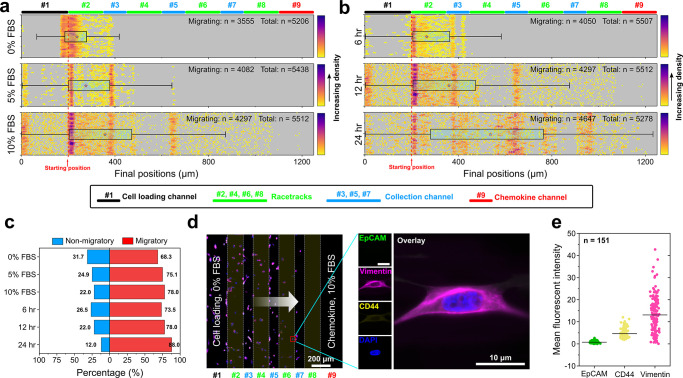
Validation of the CTC-Race assay using a clonal population of MDA-MB-231 breast cancer cells. ∼5000 cancer cells suspended in FBS-free medium were loaded into the cell-loading channel. Chemokine channel was perfused with 10% FBS at a flow rate of 0.1 μL/min. (a) Dependence of cells’ final positions on the FBS concentration. The final positions of cells at different FBS concentrations (0%, 5%, and 10%) are represented as dots, with the colors of the dots signifying their relative percentage within the cell population. The time duration of the assay was 12 h. Total numbers of seeded cells and migrating cells are shown in each subplot. Migrating cells are defined as the cells that squeeze into the racetracks and migrate toward FBS. Box plots include the smallest, lower quartile, mean, upper quartile, and largest final positions for cells. Cells’ final positions are 247.23 ± 97.98 μm (*n* = 5206) for 0% FBS, 282.04 ± 145.32 μm (*n* = 5438) for 5% FBS, and 363.94 ± 260.97 μm (*n* = 5512) for 10% FBS. All values are mean ± sd. (b) Dependence of cells’ final positions on the assay duration. Final positions of MDA-MB-231 cells in the device at different assay duration (6, 12, and 24 h) are shown. FBS concentration in the chemokine channel was 10%. Cells’ final positions are 263.23 ± 127.99 μm (*n* = 5507) for 6-h assay, 363.94 ± 260.97 μm (*n* = 5512) for 12-h assay, and 556.89 ± 303.54 μm (*n* = 5278) for 24-h assay. All values are mean ± sd. (c) Percentages of nonmigratory and migratory cells under different FBS concentrations (time = 12 h) and different assay duration (FBS concentration = 10%). (d) Left panel: an image of immunofluorescently labeled MDA-MB-231 cells in the CTC-Race device after a 24-h assay. The white arrow indicates the cells’ migration direction toward the FBS. Cells are stained with EpCAM, vimentin, CD44, and DAPI. Right panel: a migratory MDA-MB-231 cell in a single-cell racetrack expressing a high level of vimentin, medium level of CD44, and low level of EpCAM. (e) Mean EpCAM, CD44, and vimentin intensities of single migratory MDA-MB-231 cells (*n* = 151). Mean intensities of EpCAM, CD44, and vimentin are 0.76 ± 0.45, 4.60 ± 2.09, and 13.10 ± 8.10, respectively. All values are mean ± sd.

To validate the CTC-Race assay, we first characterized the migratory pattern of a clonal population of MDA-MB-231 breast cancer cells in response to a gradient of fetal bovine serum (FBS), with the goal of establishing the optimal chemokine concentration and assay duration. A typical assay is shown in Figure 1d, in which ∼5000 MDA-MB-231 cells were loaded in the cell-loading channels of the device and trapped at the entrance of single-cell racetracks. FBS-free medium in the cell-loading channels and FBS-containing medium in the chemokine channel were continuously perfused to sustain the gradient throughout the assay. The device was placed in an incubator (37 °C, 5% CO_2_) during the assay to maintain the cell viability and motility. We first investigated concentration-dependent changes in cells’ migration in response to the FBS. As illustrated in Figure 2a, we found that changes in chemokine concentrations primarily affect the percentage of the migrating population and their migratory distance. Migrating cells are defined as the cells that can squeeze into the racetracks and migrate toward higher FBS concentration. Increasing FBS concentrations from 0% to 10% resulted in an increase in the total fraction of migrating cells (68.3% to 78%) (Figure 2c), and an increase in their mean migratory distance (247.23 ± 1.36 μm for 0% FBS to 363.94 ± 3.52 μm for 10% FBS, values are shown as mean ± s.e.m) (Figure 2a). We also found that increasing assay time from 6 to 24 h resulted in an increase in the total fraction of migrating cells (from 73.5% to 88%) (Figure 2c), and an increase in their migratory distance (263.23 ± 1.72 μm for 6-h assay to 556.89 ± 4.18 μm for 24 h assay, values are shown as mean ± sem) (Figure 2b). As a result, optimized parameters including a 10% FBS in the chemokine channel and a 24-h assay time were used in subsequent cell line experiments to promote cell migration. These results highlight the function of FBS as a promigratory chemoattractant for MDA-MB-231 cells.^24,25^ Our findings also show that a small percentage of clonal MDA-MB-231 cells migrated in a random manner away from the FBS. The CTC-Race assay enables the characterization of surface antigen expressions for single migratory MDA-MB-231 cells in the device. It has been hypothesized that tumor-initiating cells arise from a biological process of phenotypic changes known as the epithelial-to-mesenchymal transition (EMT).^1,2,18,30^ During EMT, cells lose epithelial characteristics while acquiring mesenchymal traits with increased motility and elongated morphologies. To this end, MDA-MB-231 cells were immunofluorescently labeled with an epithelial marker (EpCAM), a mesenchymal marker (vimentin), a cell-migration marker (CD44), and a nucleus marker (DAPI). Marker expressions in Figure 2d,e show that on average single migrating MDA-MB-231 cells (*n* = 151) in the CTC-Race assay had a high level of vimentin expression, a medium level of CD44 expression, and a low level of EpCAM expression.

**Figure 3:**
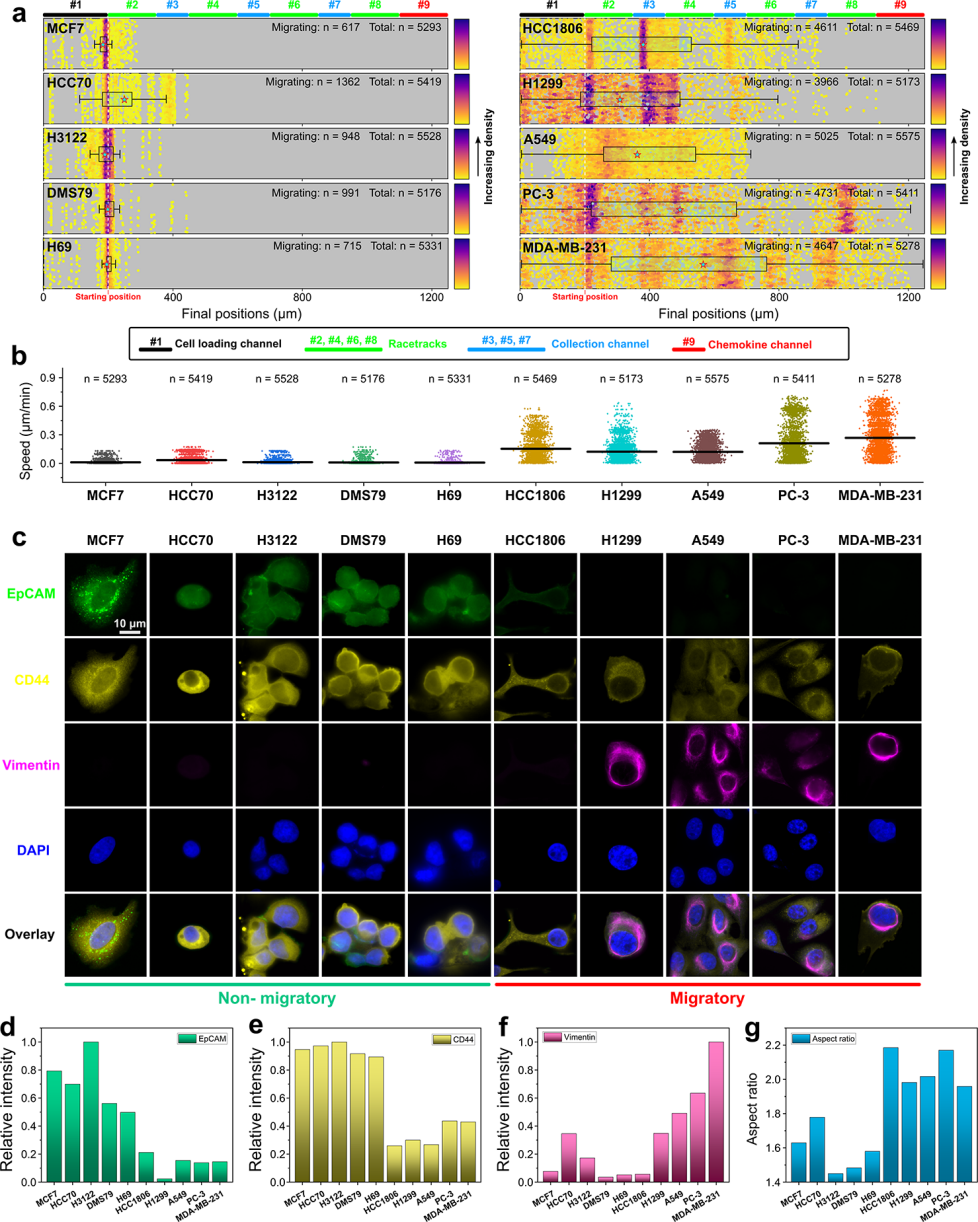
Validation of CTC-Race assay using breast, lung, and prostate cancer cell lines. ∼5000 cancer cells were loaded into the device for each cell line experiment. Optimized conditions (0.1 μL/min perfusion rate, 10% FBS concentration, and 24-h assay time) were used for all experiments. After the assay, cells were fixed and stained with four markers (EpCAM, vimentin, CD44, and DAPI). Migration distance, migration speed, cellular aspect ratio, and marker expressions of cells were analyzed from bright-field and fluorescent imaging. (a) Plots of migration distances of 10 different cancer cell lines in the CTC-Race assay (non-small-cell lung cancer, A549, H3122, H1299; small-cell lung cancer, DMS79, H69; breast cancer, HCC70, HCC1806, MCF7, and MDA-MB-231; prostate cancer, PC-3). Box plots include the smallest, lower quartile, mean, upper quartile, and largest final positions for cells. Mean final positions are 191.53 ± 33.03 μm (MCF7, *n* = 5293), 232.09 ± 77.47 μm (HCC70, *n* = 5419), 190.57 ± 33.32 μm (H3122, *n* = 5528), 199.03 ± 30.05 μm (DMS79, *n* = 5176), 198.48 ± 24.43 μm (H69, *n* = 5331), 384.76 ± 195.81 μm (HCC1806, *n* = 5469), 318.25 ± 189.51 μm (H1299, *n* = 5173), 358.96 ± 143.91 μm (A549, *n* = 5575), 467.88 ± 317.74 μm (PC-3, *n* = 5411), and 556.89 ± 303.54 (MDA-MB-231, *n* = 5278). All values are mean ± sd. (b) Migration speed of cancer cell lines. Mean speed for each cell line: 0.01 ± 0.02 μm min^–1^ (MCF7, *n* = 5293), 0.03 ± 0.05 μm min^–1^ (HCC70, *n* = 5419), 0.01 ± 0.02 μm min^–1^ (H3122, *n* = 5528), 0.01 ± 0.02 μm min^–1^ (DMS79, *n* = 5176), 0.08 ± 0.02 μm min^–1^ (H69, *n* = 5331), 0.15 ± 0.11 μm min^–1^ (HCC1806, *n* = 5469), 0.12 ± 0.10 μm min^–1^ (H1299, *n* = 5173), 0.12 ± 0.09 μm min^–1^ (A549, *n* = 5575), 0.21 ± 0.20 μm min^–1^ (PC-3, *n* = 5411), and 0.27 ± 0.19 μm min^–1^ (MDA-MB-231, *n* = 5278). All values are mean ± sd. (c) Selected immunofluorescence images of migratory cells from cell lines. Four channels are used in the immunofluorescence staining, including an epithelial maker EpCAM (green), a mesenchymal marker vimentin (magenta), a cell-migration marker CD44 (yellow), and a nucleus marker DAPI (blue). (d–f) Mean intensities (*n* = 2000) of EpCAM, vimentin, and CD44 markers across cancer cell lines. The intensities are normalized using maximal intensity (EpCAM, H3122; vimentin, MDA-MB-231; CD44, H3122). (g) Mean cellular aspect ratio of cancer cell lines (*n* = 2000).

We extended the validation of the CTC-Race assay using a total of 10 cancer cell lines, including 3 non-small-cell lung cancer cell lines (A549, H3122, H1299), 2 small-cell lung cancer lines (DMS79, H69), 4 breast cancer cell lines (HCC70, HCC1806, MCF7, MDA-MB-231), and 1 prostate cancer cell line (PC-3). The assays were conducted under optimal conditions (chemoattractant, 10% FBS; assay time, 24 h). Figure 3a summarizes the cell migration percentages and distances across cell lines. We found that five cell lines (MCF7, HCC70, H3122, DMS79, H69) had a lower percentage of cells entering the racetracks, and on average, these cells migrated a shorter distance with a slower speed compared to the other five cell lines (HCC1806, H1299, A549, PC-3, MDA-MB-231). The migration speed of individual cells was calculated from the distance migrated (difference between initial and final positions) within 24 h. Cell lines exhibited variable speed of migration during the assay time, with MCF7 cells possessing the lowest speed of 0.01 ± 0.02 μm min^–1^ (mean ± sd, *n* = 5293), and MDA-MB-231 cells possessing the highest speed of 0.27 ± 0.19 μm min^–1^ (mean ± sd, *n* = 5278) (Figure 3b). Surface antigen expressions of single migrating cells from each cell line were quantified through immunofluorescence using EpCAM, vimentin, CD44, and DAPI (Figure 3c). Analysis of 2,000 single cells’ antigen expressions in each cell line indicates that for cell lines (MCF7, HCC70, H3122, DMS79, H69) with smaller percentages of migrating cells, their EpCAM expressions were higher than the cell lines (HCC1806, H1299, A549, PC-3, MDA-MB-231) with larger percentages of migrating cells (Figure 3d). The correlation was also found to be true for the CD44 expression (Figure 3e). Vimentin expression, on the other hand, was highly expressed in high-motility cell lines (H1299, A549, PC-3, MDA-MB-231) except in the case of HCC1806 (Figure 3f). We further scrutinized the relationship between cell migration and vimentin expression in the same cell line (Figure S3). High-motility cells in the #8 racetrack had higher vimentin expression than low-motility cells in the #2 racetrack. Cell lines (HCC1806, H1299, A549, PC-3, MDA-MB-231) with a faster migratory speed and a longer migratory distance tended to have more elongated cellular morphology (Figure 3g), consistent with previous findings of mesenchymal migration.^31−33^

The CTC-Race assay is designed to minimize its impact on cells’ viability and functions to enable their downstream analyses after retrieval from the device. For this reason, the cross-sectional area of the racetracks is 150 μm^2^, larger than the ∼20 μm^2^ threshold that can induce DNA damage.^19,34^ The device is also continuously perfused with cell culture media to maintain cell viability and motilities throughout the assay. We investigated the short-term cell viability and long-term cell proliferation of H1299 lung cancer cells after their retrieval from the device at the end of the assay. Viabilities of cells retrieved from the device after 6, 12, and 24-h assay time were 98.63% ± 0.47%, 98.33 ± 0.21%, and 96.67 ± 0.63% (mean ± sd, *n* = 3), respectively, indicating a minimal impact on cell viability by the assay (Figure S4). Retrieved H1299 cells from the device continued to proliferate normally. Taken together, these data show that the CTC-Race assay can isolate the motile cancer cells and quantitively characterize the cells with biophysical and biological metrics including: (a) percentage of cells migrating in response to chemokines, (b) cell migration distance, (c) cell migration speed, (d) cell morphologies, and (e) cellular biomarker expressions, in a highly reproducible manner. The assay introduced little impact on the cells’ viability and proliferation. Migratory cancer cells can be retrieved from the assay to enable further analysis of them.

### Functional Analyses of Patient-Derived CTCs in the CTC-Race Assay

We continued validation of the CTC-Race assay using patient samples. For this validation, we conducted a study of blood samples collected from four patients exhibiting stage IIIB/IV nonsmall cell lung cancers (NSCLC) (Table S1), who were recruited and consented under an approved IRB protocol (see Methods section). CTCs from the blood samples were enriched via the inertial-FCS enrichment devices (see Methods section).^28^ Adequate numbers of CTCs (Table S1) were sorted for CTC-Race assay, possibly due to the label-free nature of the inertial-FCS devices and the advanced tumor stages of the cancer patients (Table S1).^35−37^ We note here that the inertial-FCS device is a size-based CTC isolation tool, which set a cutoff threshold size for cell isolation so that CTCs (≳15 μm in diameter) could be separated from smaller leukocytes without limiting to molecular markers for selection. While this tool allowed us to enrich adequate numbers of CTCs from blood samples for the migration study, it neglected smaller CTCs (≲15 μm in diameter) in blood samples (Figure S9). Due to the incorporation of a debris removal stage, inertial-FCS devices remove potential clusters of CTCs prior to separation (Figure S11). Other existing microfluidic technologies are better suited for CTC cluster enrichment.^38−40^ Enriched cells were divided equally into two portions, with 50% of the samples for the CTC-Race assay and the remaining 50% for cell identification through immunofluorescence. A spatial concentration gradient of growth factors including epidermal growth factor (EGF), basic fibroblast growth factor (bFGF), and fetal bovine serum (FBS) was used in the CTC-Race assay as chemoattractants to guide their migration in the single-cell racetracks, and a spatial gradient of Slit2 was used as chemorepellent to inhibit the migration of white blood cells (WBCs) that were carried over from the inertial-FCS enrichment tool.^41,42^ CTCs were seeded and allowed to migrate along the growth factors’ gradient for 24 h with incubation conditions of 37 °C and 5% CO_2_. At the end of the assay, cells were immunofluorescently labeled within the device with the epithelial marker (EpCAM), mesenchymal markers (vimentin, Vim), leukocyte marker (CD45), and nucleus staining DAPI for identification. CTCs were identified as epithelial positive (EpCAM+/Vim–/CD45–/DAPI+), mesenchymal positive (EpCAM–/Vim+/CD45–/DAPI+), or mixed epithelial and mesenchymal (EpCAM+/Vim+/CD45–/DAPI+), while WBCs were identified as EpCAM–/Vim– /CD45+/DAPI+. The migratory distance and speed of each identified CTC were imaged and calculated.

**Figure 4:**
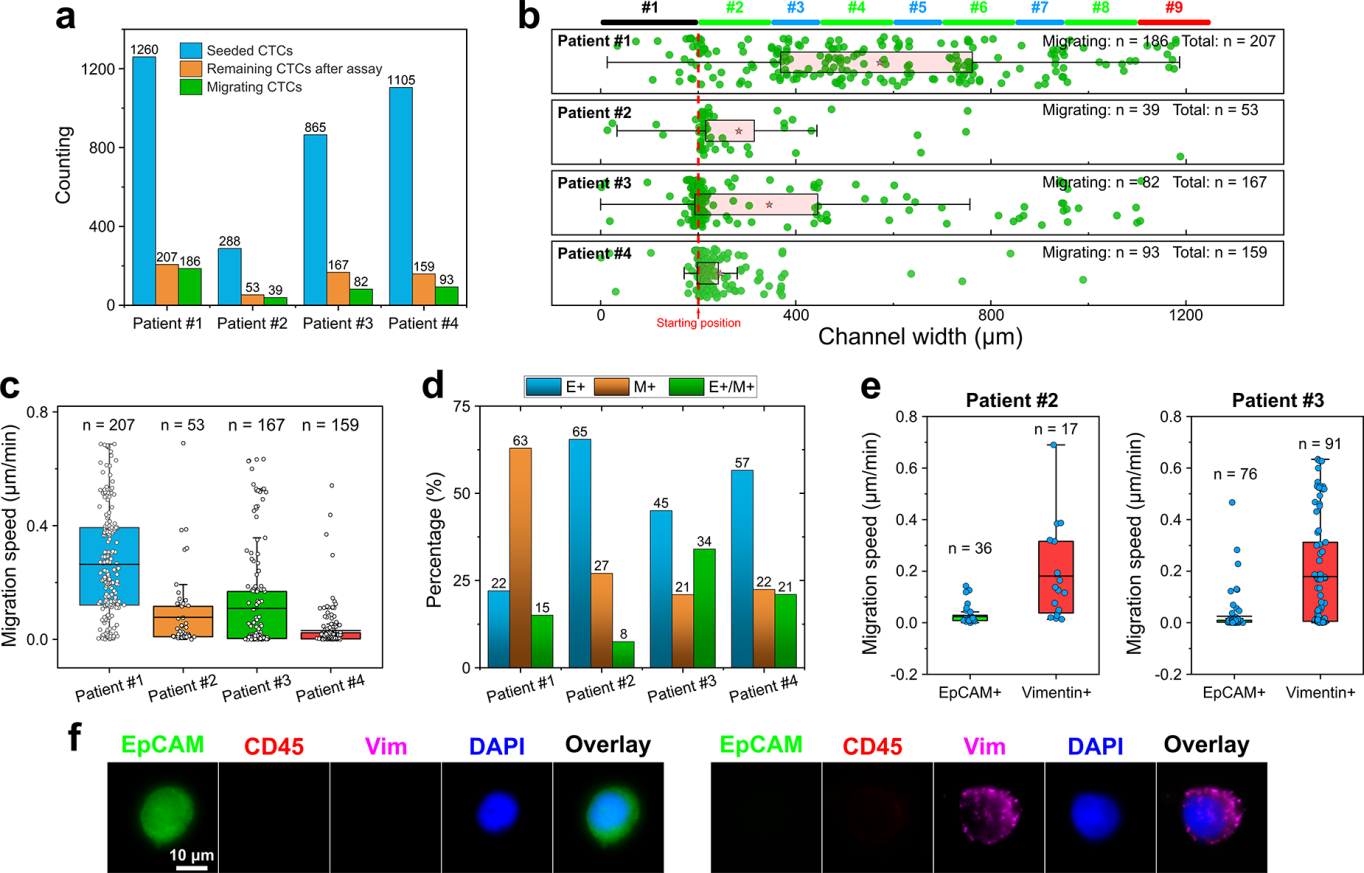
Validation of the CTC-Race assay using patient-derived CTCs. Chemokines include chemoattractants (epidermal growth factor, EGF; basic fibroblast growth factor, bFGF; fetal bovine serum, FBS) for CTCs, and chemorepellent (Slit2) for WBCs. After the assay, cells in the device were immunofluorescently labeled to determine their final positions and marker expressions. (a) Percentage of migrating CTCs in the patient samples. (b) Migration distance of CTCs in the CTC-Race assay (24 h). Final positions of cells are 564.19 ± 276.83 μm (mean ± sd, *n* = 207) for patient 1, 288.04 ± 195.78 μm (mean ± sd, *n* = 53) for patient 2, 340.28 ± 266.25 μm (mean ± sd, *n* = 167) for patient 3, and 240.08 ± 107.28 μm (mean ± sd, *n* = 159) for patient 4. (c) Migration speeds of CTCs are 0.26 ± 0.19 μm min^–1^ (mean ± sd, *n* = 207) for patient 1, 0.08 ± 0.13 μm min^–1^ (mean ± sd, *n* = 53) for patient 2, 0.11 ± 0.18 μm min^–1^ (mean ± sd, *n* = 167) for patient 3, and 0.03 ± 0.07 μm min^–1^ (mean ± sd, *n* = 159) for patient 4. (d) Biochemical phenotypes of CTCs in each patient. These CTCs were characterized through immunofluorescence (EpCAM, vimentin, CD45, and DAPI). These CTCs were not used in the CTC-Race assay. E+ indicates epithelial CTCs (EpCAM+/vimentin–/CD45–/DAPI+); M+ indicates mesenchymal CTCs (EpCAM–/vimentin+/CD45–/DAPI+); E+/M+ indicates mixed epithelial and mesenchymal CTCs (EpCAM+/vimentin+/CD45–/DAPI+). (e) Correlation between the CTCs’ migration speed in the CTC-Race assay and their biochemical phenotypes in patients 2 and 3. These CTCs were characterized through immunofluorescence (EpCAM, vimentin, and DAPI). These CTCs were used in the CTC-Race assay. The migration speeds of patient 2’s CTCs are 0.03 ± 0.04 μm min^–1^ (mean ± sd, *n* = 36, EpCAM+) and 0.18 ± 0.18 μm min^–1^ (mean ± sd, *n* = 17, vimentin+). The migration speeds of patient 3’s CTCs are 0.03 ± 0.07 μm min^–1^ (mean ± sd, *n* = 76, EpCAM+) and 0.18 ± 0.21 μm min^–1^ (mean ± sd, *n* = 91, vimentin+). (f) Selected fluorescent images of CTCs in the device (EpCAM, vimentin, CD45, and DAPI).

We found that a small percentage of the CTCs from these patient samples were able to enter single-cell racetracks and migrate toward the high concentration of growth factors/serum in the assay (Figure 4a, Table S2). For patient 1, ∼1260 estimated CTCs were seeded in the CTC-Race device at the start of the assay. At the end of the 24-h assay, we identified 207 CTCs (16.4%, 207 out of 1,260) remained in the device. Out of the remaining cells, 186 (14.8%, 186 out of 1,260) of them were considered migrating cells as they squeezed into the racetracks and moved toward the higher concentration of chemoattractants. CTCs from patients 2, 3, and 4 exhibited similar trends, with patient 2 having 13.5% (39 out of 288), patient 3 having 9.5% (82 out of 865), and patient 4 having 8.4% (93 out of 1105) migrating CTCs. CTCs that were not in the device at the end of the assay were likely apoptotic and washed away by the continuous perfusion within the assay time frame. Since the CTC-Race assay showed minimal impact on the cultured cancer cells’ viability and proliferation (Figure S4), it would be interesting to understand the reason why only a small percentage of patient-derived CTCs were able to migrate in the assay. Future viability testing of CTCs prior to the assay, as well as the studies on the likely apoptotic cells with device reconfiguration (Figures S5–S7) could shed light on this question. Figure 4b,c summarizes the CTCs’ final positions and their migration speed. Migrating CTCs exhibited variable levels of speed (Figure 4c) in each patient, with patient 1’s cells migrating at a speed of 0.26 ± 0.19 μm min^–1^ (mean ± sd, *n* = 207), patient 2’s cells migrating at a speed of 0.07 ± 0.13 μm min^–1^ (mean ± sd, *n* = 53), patient 3′s cells migrating at a speed of 0.11 ± 0.18 μm min^–1^ (mean ± sd, *n* = 167), and patient 4’ cells migrating at a speed of 0.03 ± 0.07 μm min^–1^ (mean ± sd, *n* = 159). To understand the varying migration distances and speeds of CTCs between the patients, we studied the biochemical phenotypic subtypes of CTCs in each patient, which is summarized in Figure 4d, which shows an interesting comparison between the patients. CTCs were immunofluorescently labeled with EpCAM, vimentin, CD45, and DAPI (Figure 4f). We found that enriched CTCs of patient 1 had a significant portion of mesenchymal phenotype while the majority of patient 2, 3, and 4’s CTCs were predominately epithelial. The detailed distributions of CTC subtypes were the following: patient 1 (epithelial 22%, mesenchymal 63%, and mixed epithelial and mesenchymal 15%), patient 2 (epithelial 65%, mesenchymal 27%, and mixed epithelial and mesenchymal 8%), patient 3 (epithelial 45%, mesenchymal 21%, and mixed epithelial and mesenchymal 34%), and patient 4 (epithelial 57%, mesenchymal 22%, and mixed epithelial and mesenchymal 21%). We further studied the migration speed difference between epithelial and mesenchymal CTCs in Figure 4e. Migrating CTCs from patients 2 and 3 were immunofluorescently labeled with EpCAM and vimentin in the device after the 24-h assay. For both patients 2 and 3, their mesenchymal CTCs appeared to migrate at a faster speed compared to the epithelial CTCs. Specifically, the migration speed of patient 2’s epithelial CTCs was 0.03 ± 0.04 μm min^–1^ (mean ± sd, *n* = 36, EpCAM+), and mesenchymal CTCs was 0.18 ± 0.18 μm min^–1^ (mean ± sd, *n* = 17, vimentin+). The migration speed of patient 3′s epithelial CTCs was 0.03 ± 0.07 μm min^–1^ (mean ± sd, *n* = 76, EpCAM+), and mesenchymal CTCs was 0.18 ± 0.21 μm min^–1^ (mean ± sd, *n* = 91, vimentin+). Taken together, due to the predominate percentage of mesenchymal phenotype in patient 1’s CTCs, and the predominate percentages of epithelial phenotype in patients 2, 3, and 4’s CTCs, patient 1’s cells migrated at a higher speed of 0.26 ± 0.19 μm min^–1^ (mean ± sd, *n* = 207) than patient 2’s cells of 0.07 ± 0.13 μm min^–1^ (mean ± sd, *n* = 53), patient 3’s cells of 0.11 ± 0.18 μm min^–1^ (mean ± sd, *n* = 167) and patient 4’s cells of 0.03 ± 0.07 μm min^–1^ (mean ± sd, *n* = 159) as shown in Figure 4c. These preliminary data confirm that the CTC-Race assay can isolate the subpopulations of migrating CTCs from patient samples and characterize their biological and biophysical properties.

### Connection between CTC Motility and Clinical/Genetic Information

**Figure 5:**
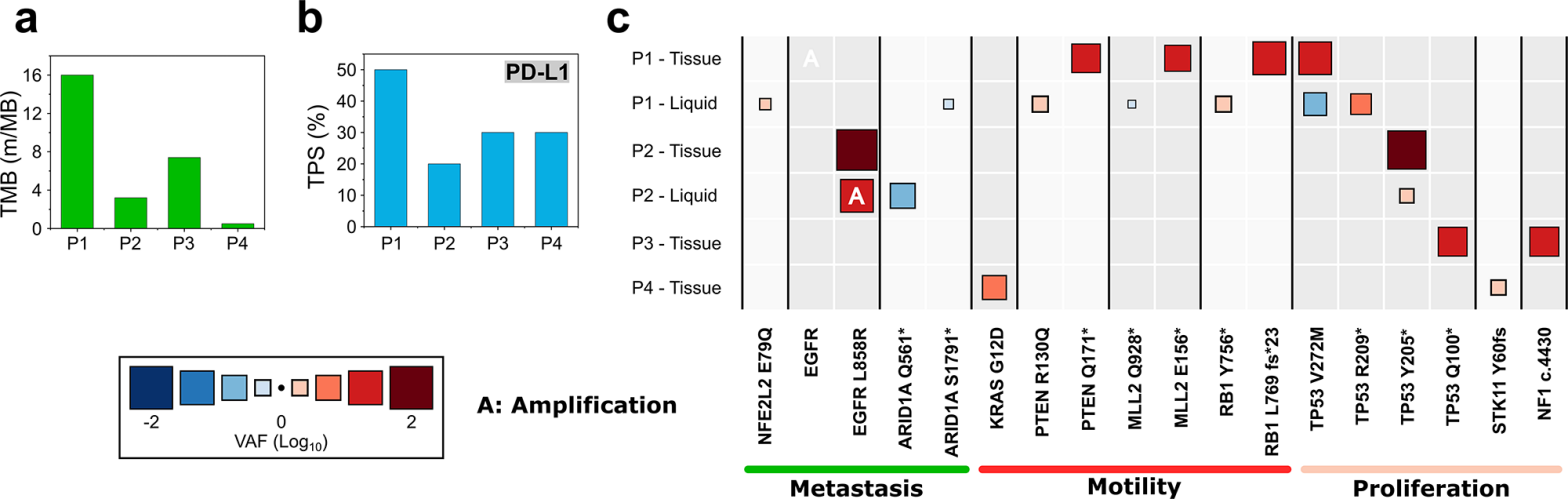
Genomic information on tissue and blood biopsy of cancer patients. (a) Tumor mutational burden (TMB) of cancer patients. (b) PD-L1 expression of cancer patients evaluated with tumor proportion score (TPS). (c) Heat map of variant allele frequency (VAF) of cancer patients with either tissue or blood biopsy. The size and color gradient of the box represent the VAF value. Patient 1’s tissue biopsy VAF values are PTEN Q171* (15.3%), MLL2 E156* (12.1%), RB1 L769 fs*23 (28.2%), TP53 V272 M (25.7%), and EGFR amplification. Patient 1’s blood biopsy VAF values are NFE2L2 E79Q (2.3%), ARID1A S1791 (0.61%), PTEN R130Q (3.1%), MLL2 Q928* (0.81%), RB1 Y756* (3.1%), TP53 V272 M (0.22%), and TP53 R209* (3.8%). Patient 2’s tissue biopsy VAF values are EGFR L858R (64.6%) and TP53 Y205* (49.9%). Patient 2’s blood biopsy VAF values are EGFR L858R (27.1%), ARID1A Q561* (0.1%), TP53 Y205* (2.8%), and EGFR amplification. Patient 3’s tissue biopsy VAF values are TP53 Q100* (15.9%) and NF1 c.4430 (16.6%). Patient 4’s tissue biopsy VAF values are KRAS G12D (7.2%) and STK11 Y60 fs (2.5%).

All four NSCLC patients who donated their blood samples for the CTC-Race assay were diagnosed with late-stage (stage IIIB-IV) metastatic cancers (Tables S1 and S3) at the time of enrollment. We accessed the genetic information on each patient’s tumor tissue and blood biopsies (Figure 5, Table S3) and compared them to the functional data (migration and epithelial/mesenchymal phenotypes) of CTCs obtained from the CTC-Race assay. Patient 1 exhibited the highest tumor mutational burden (TMB) (Figure 5a) and the highest expression of programmed death-ligand 1 (PD-L1) in his tumor tissue (Figure 5b). High TMB is indicative of a greater frequency of genetic mutations within tumor cells,^43^ while PD-L1 is involved in the regulation of cancer cell migration and invasion.^44,45^ Interestingly, CTCs from patient 1 were predominately mesenchymal phenotype and migrated in the CTC-Race assay with the fastest speed among all patients. However, we note here that TMB and PD-L1 expression measurements were performed in the patient’s tumor tissue, which did not represent the tumor cells that moved into blood circulation. Future studies should focus on understanding the connections between internal cellular programs and the observed migration from CTC-Race using the same clone of cells. Analysis of the variant allele frequency (VAF) of genes associated with metastasis, motility, and proliferation was also conducted to understand the heterogeneity of gene mutations among the patients (Figure 5c). We observed that patient 1’s tissue biopsy showed amplified VAF values indicative of cell motility, while the blood biopsy showed amplified VAF values indicative of metastasis, cell motility, and proliferation. The limitations of this preliminary study with a small cohort of patients preclude statistical and clinical significance; it does highlight the potential of utilizing the CTC-Race assay to study connections between CTC functions and cellular programs. An interesting study from a biological perspective, which was not conducted in this work due to limited resources, is to understand the connection between the expression of genes and transcripts of patient-derived CTCs and their motility from the CTC-Race assay. In summary, the CTC-Race assay was demonstrated here to be able to isolate migratory tumor cells from a heterogeneous cell population with high purity. These migratory CTCs can be extracted from the device based on their levels of migration due to the design of the device (Figure 1d). These features of the CTC-Race assay could enable concurrent single-CTC functional, genomic, transcriptomic, and proteomic analyses to provide insights into cancer metastasis.

## Conclusion

Studying the migratory characteristics of CTCs presents a way to identify cells with a greater tumor-initiating capacity and may increase our understanding of the metastatic process. However, CTCs are extremely rare and highly heterogeneous in patient samples. For this reason, existing microfluidic methods for cancer cells’ migration studies were conducted with cultured cells^23−26^ or cells from xenografted tumors.^27^ Phenotyping based on the motility of patient-derived CTCs has not been achieved.^4^ We presented the CTC-Race assay, which is a microfluidic platform with 5000 single-cell racetracks and sustained chemokine gradients that can not only isolate migratory CTCs from patient samples based on their own motility but also quantitatively study the biological and biophysical characteristics of these motile cells with single-cell resolution, including cell migration distance/speed, cellular morphology, and biomarker expressions. The CTC-Race assay can accommodate single-cell studies from just a few cells to up to 5000 cells simultaneously (Figure S8). The assay was validated using both cultured cancer cells and enriched CTCs from four metastatic NSCLC patients with advanced tumor stages that resulted in a large number of tumor cells in their blood circulation. In processing patient-derived CTCs, the CTC-Race assay was able to isolate a small percentage of tumor cells (11.5% of the whole CTC population, *n* = 4) that were migratory from a heterogeneous cell population with high purity (97.85%, *n* = 4, Table S2) and characterize the cells’ migrating distance/speed and relevant biomarker expressions. Migratory cells from the assay can be retrieved from the device and are amenable to downstream analyses. The CTC-Race assay can preserve nonmotile and/or apoptotic CTCs from the patient samples with reconfiguration of chemokines profiles within the device. Preliminary study with four metastatic NSCLC patients showed a connection between the CTC’s functions to genetic mutations in their tissue and blood biopsy samples. In summary, we demonstrated a motility study of patient-derived CTCs using the microfluidic CTC-Race assay. One of the next steps to validate CTC-Race uses low numbers of CTCs from early-stage cancer patient samples. With further investigations, the CTC-Race assay could be used to study the connections between expression of genes and transcripts of patient-derived CTCs and their motility that provide insights into cancer metastasis.

## Methods

### CTC-Race Device Fabrication

The microfluidic device of the CTC-Race assay contains nine main channels (two cell-loading, one chemokine channel, and six cell-collection channels) and eight single-cell racetrack segments. A single device contains 5000 single-cell racetrack channels (2500 tracks on each side). The main channels had a height of 50 μm, and the racetracks had a height of 5 μm. The device’s mold was microfabricated on a silicon wafer (WaferPro, Santa Clara, CA) with SU-8 3005 and 2025 photoresists (MicroChem, Westborough, MA). Polydimethylsiloxane (PDMS, Dow Corning, Midland, MI) was used to replicate the SU-8 mold and bonded on a glass slide to obtain the final device. Before each use, devices were immersed in 70% ethanol and disinfected under UV light for 12 h. Ethanol was removed by air-drying in a biosafety cabinet. Channels’ surfaces were coated with 50 μg/mL type 1 collagen solution (Advanced Biomatrix, San Diego, CA) following the manufacturer’s recommended protocol. The coated devices were stored at 4 °C.

### Cell Lines

Ten human cancer cell lines including four breast cancer cell lines (MCF7, MDA-MB-231, HCC1806, and HCC70), three non-small-cell lung cancer (NSCLC) cell lines (A549, H1299, H3122), two small-cell lung cancer (SCLC) cell lines (DMS79 and H59), and one prostate cancer cell line (PC-3) were purchased from ATCC (Manassas, VA). STR profiling information on these cell lines is provided in the Supporting Information (Table S4). Cell cultures followed the manufacturing instructions. Breast cancer cell lines MCF7 and MDA-MB-231 were cultured in DMEM medium (Thermo Fisher Scientific, Waltham, MA), and the other cell lines were cultured in RPM 1640 medium (Thermo Fisher Scientific, Waltham, MA). DMEM and RPMI medium were supplemented with 10% (v/v) fetal bovine serum (FBS, Thermo Fisher Scientific, Waltham, MA), 1% (v/v) penicillin/streptomycin solution (Thermo Fisher Scientific, Waltham, MA), and 0.1 mM nonessential amino acid (NEAA, Thermo Fisher Scientific, Waltham, MA). All of the cell lines were cultured at 37 °C with 5% CO_2_. Cells were released with 0.05% trypsin-EDTA solution (Thermo Fisher Scientific, Waltham, MA), centrifugated (5 min, 500 g) to remove the supernatant, and resuspend in 1× Dulbecco’s phosphate buffered saline (DPBS, Thermo Fisher Scientific, Waltham, MA). To track the cell trajectories in the i^2^ FCS device, cells were either stained with 3 μM CellTracker Green or 3 μM CellTracker Orange (Thermo Fisher Scientific, Waltham, MA) for 30 min at 37 °C and then washed and resuspended with culture medium. Cells were counted with Countess 2 (Thermo Fisher Scientific, Waltham, MA) and diluted to 1×10^4^ cells/mL in culture medium. After dilution, the exact number of cells was confirmed with a Nageotte counting chamber (Hausser Scientific, Horsham, PA). Variable numbers (10, 50, 100, and 200) of cancer cells were spiked into 0.015% (v/v) ferrofluid for spiking experiments.

### Patient Recruitment and Blood Donation

Non-small-cell lung cancer (NSCLC) patients were recruited with informed consent at the University Cancer and Blood Center, LLC (Athens, GA), and their blood samples were obtained following a protocol approved by the Institutional Review Board (IRB) at the University of Georgia (VERSION00000869). Patient information is given in Table S1.

### CTC Enrichment from Blood Samples

A previously reported CTC enrichment tool (inertial-ferrohydrodynamic cell separation,^28^ inertial-FCS) was used for label-free isolation of circulating cancer cells in cancer patients’ blood samples. Blood samples from NSCLC patients were processed by the inertial-FCS devices within 40 min of blood draws. The total time of the inertial-FCS processing time of blood samples was ∼10 min. After CTC enrichment by the inertial-FCS devices, isolated cells were seeded into CTC-Race devices within ∼20 min after enrichment. Blood was drawn from the patients and processed by the inertial-FCS devices, which used a combination of inertial focusing and ferrohydrodynamic separation to separate large cancer cells from smaller blood cells. The inertial-FCS devices were first treated with ethanol (70%) flushing for 10 min. The microchannels were then primed with PBS supplemented with 0.5% (w/v) bovine serum albumin (BSA) and 2 mM EDTA (Thermo Fisher Scientific, Waltham, MA). Blood samples were first lysed with RBC lysis buffer (eBioscience, San Diego, CA) for 10 min at room temperature, centrifuged at 500 *g* for 5 min, and resuspended in a 0.05% (v/v) ferrofluid. Sample fluids and sheath ferrofluids were individually controlled with syringe pumps (Chemyx, Stafford, TX) at variable flow rates during sample processing. After processing with the inertial-FCS device, collected cells were centrifuged at 500 *g* for 5 min at room temperature and resuspended in DMEM/F12 medium supplemented with B27 supplement (1×; Thermo Fisher Scientific, Waltham, MA), epidermal growth factor (20 ng/mL; Millipore Sigma, Burlington, MA), and basic fibroblast growth factor (10 ng/mL; Thermo Fisher Scientific, Waltham, MA), l-glutamine (2 mM; Thermo Fisher Scientific, Waltham, MA), and penicillin–streptomycin (1X; Thermo Fisher Scientific, Waltham, MA). The number of CTCs was counted with immunofluorescence staining following the established protocol.^28^ Briefly, isolated cells were identified with CTC markers (EpCAM, vimentin), leukocyte marker (CD45), and nuclei marker (DAPI). The number of seeded CTCs in the CTC-Race assay was estimated based on the CTC counts obtained here.

### CTC-Race Assay

#### Cancer Cell Lines

The CTC-Race device was filled with FBS-free cell culture medium (DMEM or RPMI medium supplemented with 1% (v/v) penicillin/streptomycin and 0.1 mM nonessential amino acid and incubated at 37 °C for 1 h. Cell-collection channel inlets and outlets were sealed before cell loading. Cancer cells, collected from culture plates with 0.25% Trypsin-EDTA (ThermoFisher, Waltham, MA) and pelleted by centrifugation at 500 *g* for 5 min, were resuspended in FBS-free culture medium (DMEM or RPMI), to a concentration of 5 × 10^5^ cells/mL. 50 μL aliquot of cell suspension was pipetted into the cell-loading channels. The device was then placed in the incubator (37 °C, 5% CO_2_) for 40 min to ensure the cell adhesion to the glass side of the channel. FBS-free medium was flowed into the cell loading channel, while a medium containing 10% FBS was flowed into the chemokine channel. The flow rates of the medium with or without FBS were both 0.1 μL/min. For the duration of the assay, the device was placed in an incubator (37 °C and 5% CO_2_).

#### Patient-Derived CTCs

The CTC-Race device was filled with chemokine-free DMEM/F12 medium (supplemented with B27 supplement, 2 mM l-glutamine, and 1% (v/v) penicillin–streptomycin) and incubated at 37 °C for 1 h. Cell-collection channels’ inlets and outlets were sealed before cell loading. Enriched patient-derived CTCs were resuspended in 100–200 μL of chemokine-free DMEM/F12 medium. 50 μL of cell suspension was pipetted into the cell-loading channels. To maximize cell loading efficiency, the outlets/inlets of the cell-collection channels, outlets of cell-loading channels, and the inlet of the chemokine channel were sealed, and a 1 mL syringe (BD, Franklin Lakes, NJ) was connected to the outlet of the chemokine channel. Cells were continuously seeded into the channel and trapped near the single-cell racetracks with minimum loss using a withdrawal model at a flow rate of 10 μL min^–1^. The device was then placed in the incubator (37 °C, 5% CO_2_) for 40 min to ensure the cell adhesion to the glass side of the channel. Chemokine-free DMEM/F12 medium was flowed into the cell-loading channel, while a DMEM/F12 medium containing 10% FBS, 20 ng/mL epidermal growth factor, and 10 ng/mL basic fibroblast growth factor was flowed into the chemokine channel. The flow rates of medium with or without FBS were both 0.1 μL/min. For the duration of the assay, the device was placed in an incubator (37 °C, 5% CO_2_).

### Cell Retrieval from the CTC-Race Assay

To retrieve cells from the CTC-Race assay device, 0.25% trypsin–EDTA was flushed into the device for 5 min at 37 °C. Cells were then flushed out by cell culture medium and collected into the 48-well plate (Corning, Corning, NY).

### Cell Viability and Proliferation Assays

For viability assay, cell viability assays were performed after 6, 12, and 24-h assay using Live/Dead viability/cytotoxicity kit (ThermoFisher, Waltham, MA). Medium in the device was replaced with a working solution (2 μM calcein-AM and 4 μM propidium iodide (PI) in D-PBS) for 35 min at room temperature. Cells were then observed and counted under an inverted microscope (Carl Zeiss, Germany). For proliferation assay, cancer cells retrieved from device were cultured in a 48-well plate with an appropriate medium, and the medium was refreshed every 24 h during the first 3 days. The number of cells and cellular morphology were inspected at 0 and 72 h. After 72 h, Live/Dead assays were performed using Live/Dead viability/cytotoxicity kit following manufacturer’s protocol.

### Immunofluorescence in the CTC-Race Device

After the CTC-Race assay, cells in the device were fixed with 4% (w/v) PFA (Santa Cruz Biotechnology, Dallas, TX) for 10 min and subsequently permeabilized with 0.1% (v/v) Triton X-100 (Alfa Aesar, Haverhill, MA) in PBS for 10 min. Blocking buffer (Santa Cruz Biotechnology, Dallas, TX) was applied for 30 min to block the nonspecific binding sites of cells. Cells were then stained with primary antibodies including anti-EpCAM, anti-CD44, and anti-vimentin (Santa Cruz Biotechnology, Dallas, TX) at 4 °C overnight. Stained cells were washed with PBS and covered with DAPI-Fluoromount (Electron Microscopy Sciences, Hatfield, PA) before fluorescent imaging. Leukocyte marker (CD45, Abcam, Cambridge, MA) was used when the sample contained WBCs.

### Cell Migration and Morphology Characterization

The cell aspect ratio was calculated by the ratio of their major axis to the minor axis. Cell marker expressions were calculated by the sum of the pixel intensities divided by the number of pixels corresponding to each cell. The protocol of the cellular migration distance and speed calculation is described as follows. After cell seeding in the incubator (3 7°C, 5% CO_2_) for 40 min to ensure the cell adhesion to the glass side of the channel, a bright-field snapshot of the cells within the device was taken at time = 0 h to ensure that the cells were seeded at the entrances of the migration channels (starting position labeled in Figure S12a, red dashed line indicating the starting positions of the cells). Afterward, medium was flowed into the cell loading channel and the chemokine channel. For the total duration of the assay (up to 24 h), the device was placed in an incubator (37 °C, 5% CO_2_). At the end of the assay, the device was taken out of the incubator, and cells within the device were imaged again in bright-field mode. Cells were then fluorescently stained with DAPI to determine the nucleus location. Using the locations of cells (nucleus of the cell in the circles of Figure S12b) within the device in the images at the end of the assay, we calculated the total migration distance of the cells to be the straight-line difference between starting points and nuclei of cells (red dashed lines in Figure S12b). The migration speed of the cells is defined as the total migration distance divided by the total assay time. In the above calculations, two assumptions were made: (1) All cells start their migration from the starting positions indicated in Figure S12a. This assumption was validated by the images captured at assay time *t* = 0 where all cells could be clearly seen at the starting position (entrances of migration channels). (2) Cells migrated in a straight-line manner from the starting points to their final locations at the end of assay. The straight line is perpendicular to the direction of the cell collection channel. There are instances where cells could enter different (non-straight-line) migration channels during their movement toward the chemokine gradient which could introduce a systemic underestimate of the cellular migration distance and speed.

### Genetic Information Collection

The tissue biopsy and blood biopsy of cancer patients were conducted at the University Cancer and Blood Center, LLC (Athens, GA). Biopsy locations for each patient are listed in Table S3. Tissue biopsy and blood biopsy samples were sent to Tempus and Foundation Medicine for genomic sequencing. Genomic data including tumor mutational burden (TMB), tumor proportion score (TPS), and variant allele frequency (VAF) for each sample were analyzed and summarized by the personnel at the University Cancer and Blood Center, LLC (Athens, GA).

## Supporting Information Available

Dependence of cells’ final position on the coating of the channel surface; chemokine gradient study; mean vimentin intensities of cells in the racetracks; cell viability and proliferation assay of H1299 cells; separation of migratory cells from nonmigratory cells using the CTC-Race device under optimized conditions; effects of chemokines on white blood cells (WBCs) in the CTC-Race assay; reconfiguration of CTC-Race assay to preserve nonmotile cancer cells; CTC-Race assay with a variable number of starting cells; diameter distribution of cultured cancer cell lines and patient-derived CTCs; relationship between mean cell diameter and mean cell migration distance for cultured cancer cell lines; schematic of the inertial-FCS cell sorting device; protocol of tracking single cells in the migration channels of the CTC-Race device and the method of calculating migration distance and speed of cells; patient information and CTC isolated from each patient; purity of motile CTCs from patient samples in the CTC-Race assay; additional clinical information on patients; STR profile of cancer cell lines used in this study (PDF)
