# Supplementary material for: CTC-Race: Single-Cell Motility Assay of Circulating Tumor Cells from Metastatic Lung Cancer Patients
Source: ACS Nano. 2024 Mar 11;18(12):8683–93. doi: 10.1021/acsnano.3c09450 (PMC10976960; doi:10.1021/acsnano.3c09450)
Supplement: Supplementary file 1 — nn3c09450_si_001.pdf [file nn3c09450_si_001.pdf]

Supplementary Information

CTC-Race: single-cell motility assay of circulating tumor cells from metastatic lung cancer patients

Yang Liu,[a] Wujun Zhao,[b] Jamie Hodgson,[c] Mary Egan,[c] Christen N. Cooper Pope,[c] Glenda Hicks,[c] Petros G. Nikolinakos,[c] and Leidong Mao*[d]

[a]School of Chemical, Materials and Biomedical Engineering, College of Engineering, The University of Georgia, Athens, Georgia 30602, USA

[b]FCS Technology, LLC, Athens, Georgia 30602, USA

[c]University Cancer and Blood Center, LLC, Athens, Georgia 30607, USA

[d]School of Electrical and Computer Engineering, College of Engineering, The University of Georgia, Athens, Georgia 30602, USA

*Email: Yang Liu (liuy@uga.edu), Leidong Mao (mao@uga.edu)

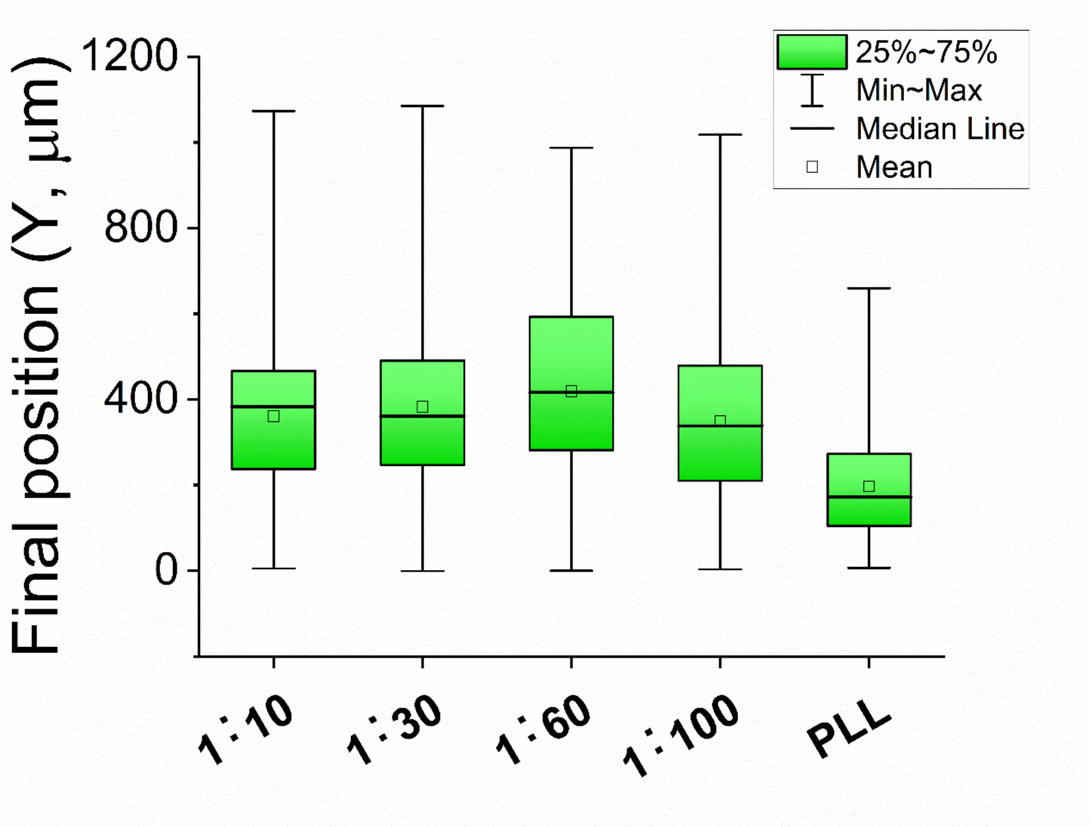

Figure S1. Dependence of cells' final position on the coating of the channel surface. The channels were coated with collagen type I of variable concentrations, and with poly-L-lysine. MDA-MB-231 cells were used for this study. Other experimental conditions are: 10% FBS, 12-hour assay, and 0.1 µL/min perfusion flow rate. Collagen type I diluted by 60 times (50 µg min^{-1}) resulted in the largest migration distance and was used in the CTC-Race device.

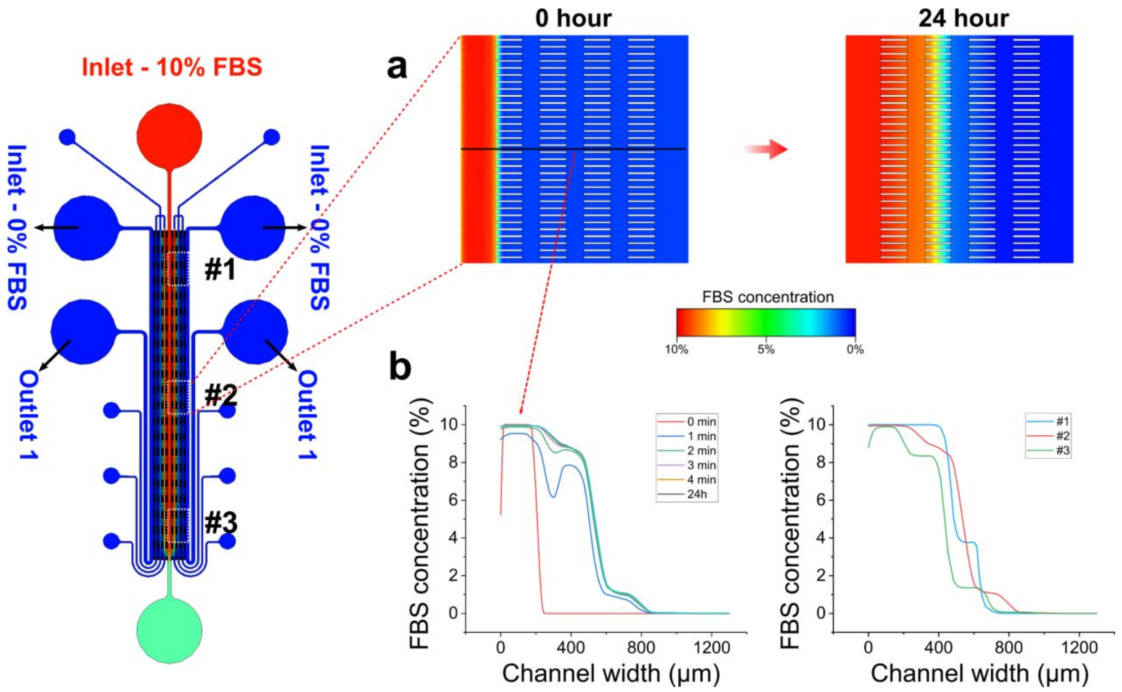

Figure S2. Chemokine gradient study. **a** Simulation of chemokine concentration gradient in the CTC-Race device. The diffusion coefficient of FBS is 61 $\mu m^2/s$. Viscosity of the medium is 0.94 cP. Flow rate of perfusion is 0.1 μL/min. A gradient of the chemokine can be established for 24 hours in the device. **b** Left: line plots show that the chemokine gradient can be established within 4 minutes after the start of the perfusion and sustained for 24 hours. Right: the chemokine gradient is consistent across the device. Observation windows labeled with #1, #2, and #3 are in the left, middle and right parts of the device.

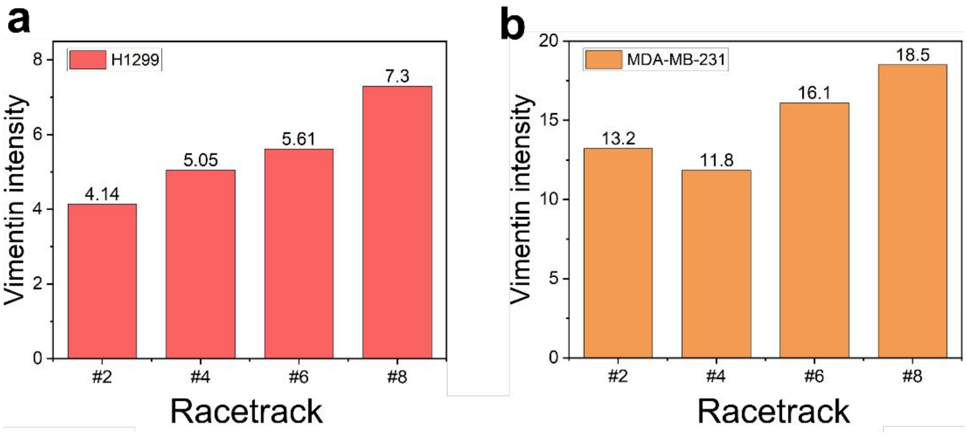

Figure S3. Mean vimentin intensities of cells in the Racetracks (#2, #4, #6, and #8) **(a)** Mean vimentin intensity of human lung cancer cell line (H1299). **(b)** Mean vimentin intensity of human breast cancer cell line (MDA-MB-231).

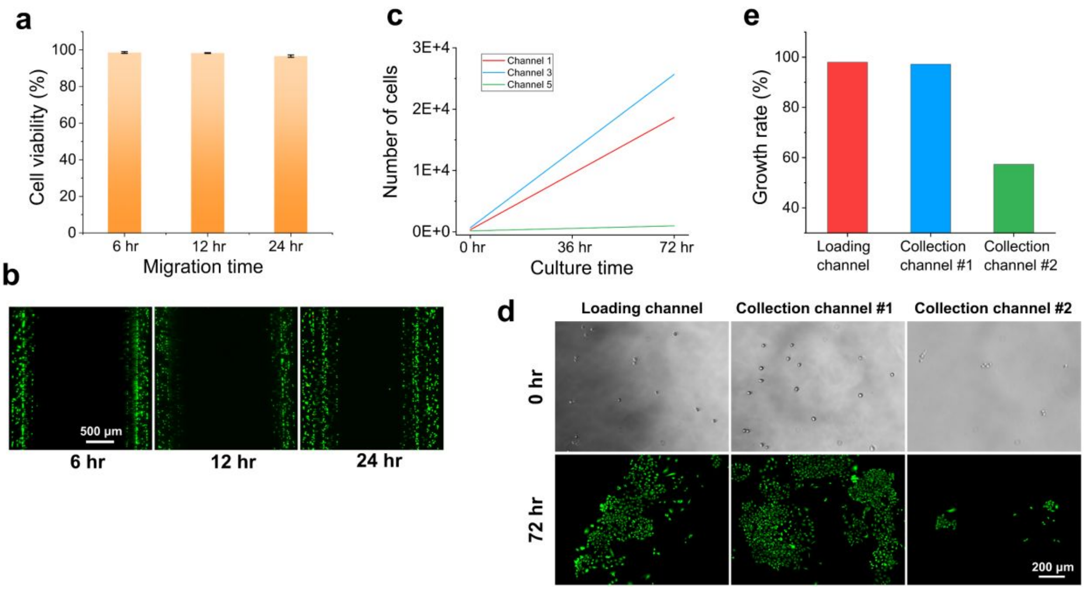

Figure S4. Cell viability and proliferation assay of H1299 cells. **a-b** Short term viability of H1299 cells after 6 hours, 12 hours and 24 hours' assay time under optimized conditions (flow rate = 0.1 μL/min, FBS concentration = 10%). **a** Cell viability of H1299 cells at 6 hours, 12 hours and 24 hours' assay was determined to be 98.63% ± 0.47%, 98.33 ± 0.21% and 96.67 ± 0.63% (mean ± s.d.), respectively (n = 3). **b** Representative images of Live/Dead staining of H1299 cells in the device. **c-e** Cell proliferation assay. **C** Representative images of retrieved H1299 cell culture over a 72-hour period. Live/Dead staining of the culture cells shows viable cells. **d** Total number of cells from cell culture. Cells with different migration distance were collected. **e** Growth rate of retrieved cells with different migration distance. Growth rate is calculated by (cell number at 72 hr – cell number at 0 hr)/cell number at 72 hr.

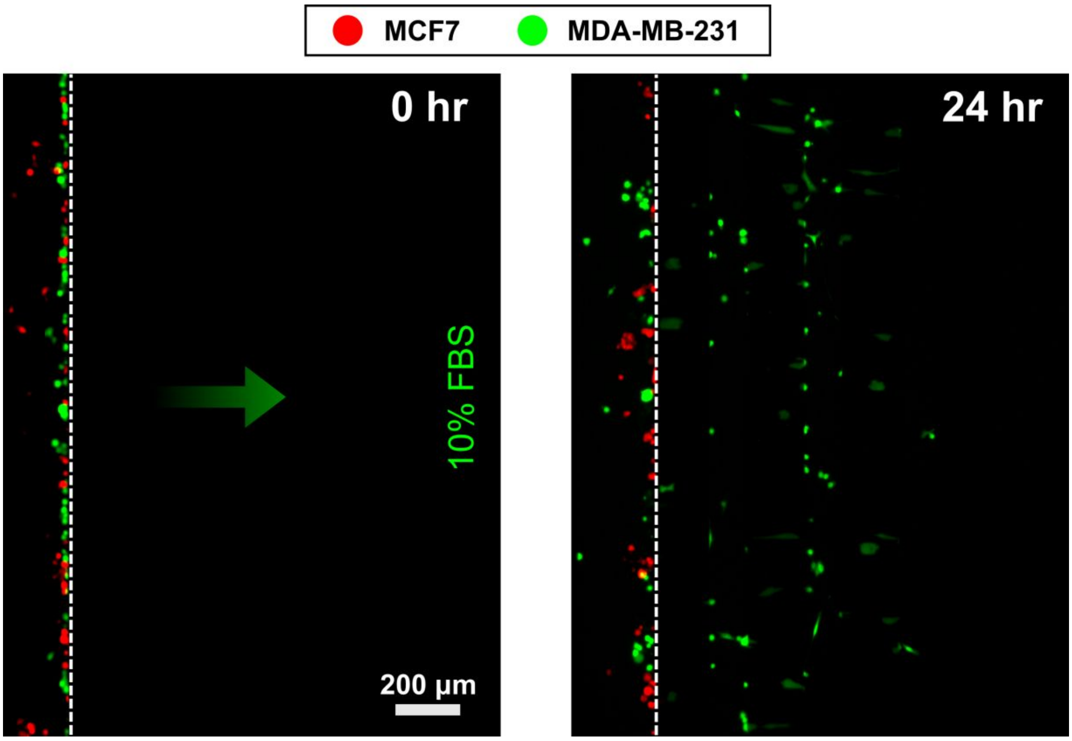

Figure S5. Separation of migratory cells (green, MDA-MB-231) from non-migratory cells (red, MCF7) using the CTC-Race device under optimized conditions (perfusion flow rate = 0.1 μL/min, FBS concentration = 10%, and t = 24 hour). The green arrow indicates migration direction. White dash line represents the starting point of the single-cell racetracks.

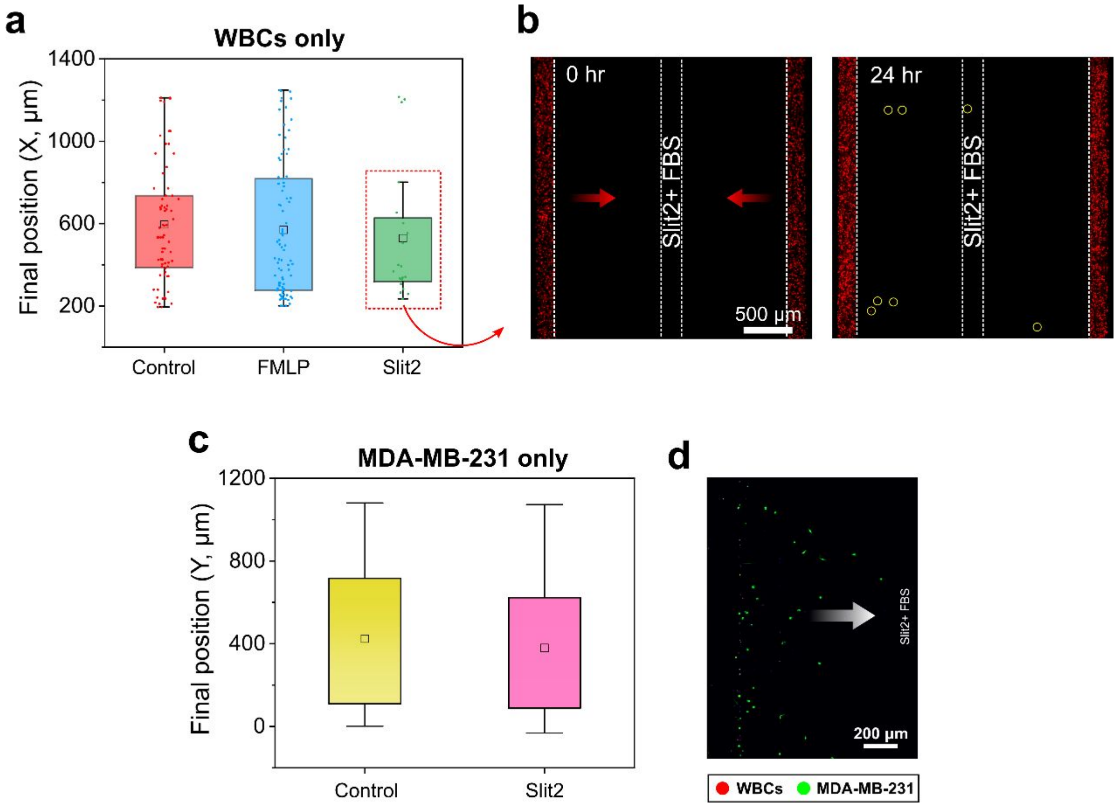

Figure S6. Effects of chemokines on white blood cells (WBCs) in the CTC-Race assay. **a** WBCs (n=~5000) migration assays under control (FBS only), chemoattractant (FMLP) and chemorepellent (Slit2). FBS (10%) or Slit2 (5 µg/mL) were loaded into the chemokine channel, while FMLP (200 ng/mL) were loaded in the cell-loading channel. The plot shows positions of individual WBCs and the rectangular symbols indicate the mean values. FMLP had less impact on the migration of WBCs while Slit2 could significantly inhibit the migration of WBCs. **b** WBCs migration is inhibited using Slit2 (5 µg/mL) in 10% FBS. Assay time is 24 hours. Perfusion flow rate was 0.1 µL/min. WBCs were stained with cell tracker red. Fluorescence images were taken at 0 hour and 24 hours. Yellow circles indicate the migratory WBCs. **c** Effects of Slit2 (5 µg/mL in 10% FBS) on MDA-MB-231 breast cancer cells. Perfusion flow rate was 0.1 µL/min. The plot shows positions of MDA-MB-231 and the rectangular symbols indicates the mean value. Slit2 had a minimal impact on the migration of MDA-MB-231. **d** WBCs (red) and MDA-MB-231 (green) mixture (1:1) migration assay with chemokine channel loaded with 10% FBS and Slit2 (5 µg/mL). After 24 hours, majority of MDA-MB-231 cells moved into the single-cell racetracks with minimal WBCs contamination.

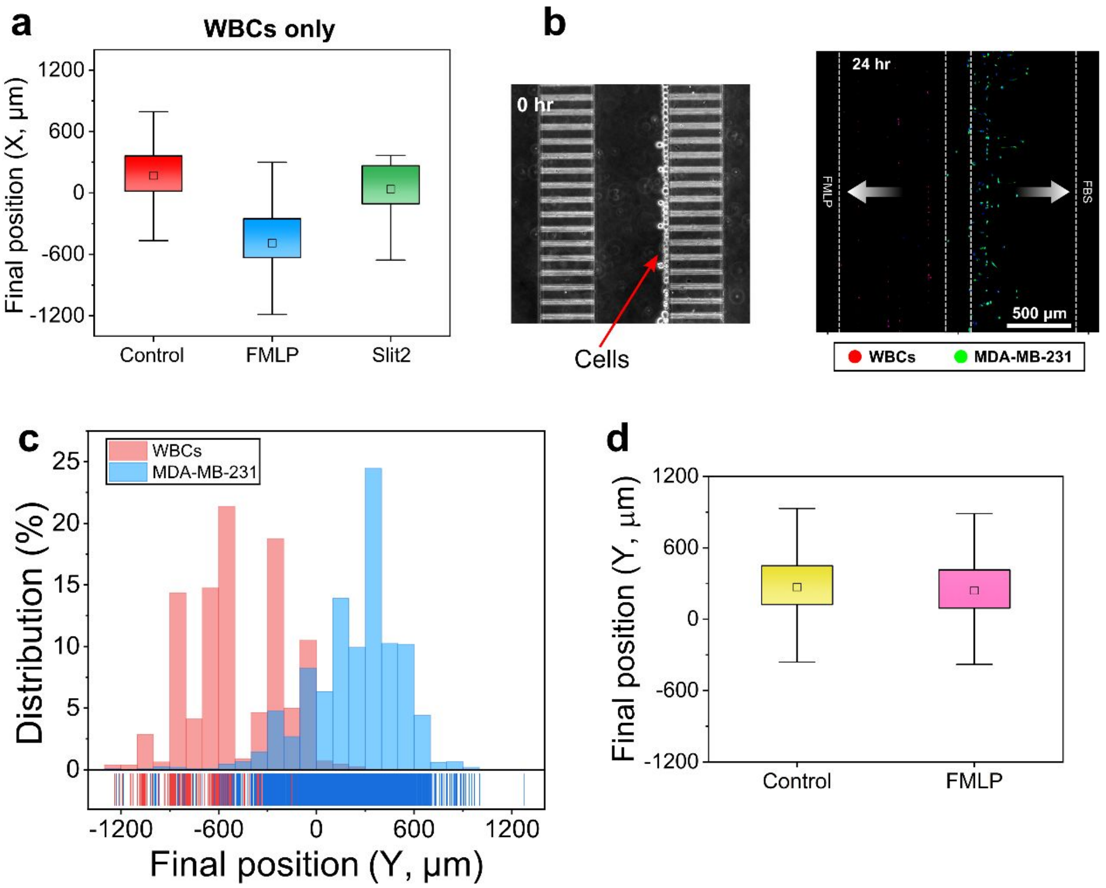

Figure S7. Reconfiguration of CTC-Race assay to preserve non-motile cancer cells. **a** WBCs migration assay using chemokines including FBS, FMLP and Slit2. WBCs (n = ~2500) were loaded into the channel that was previously used for chemokine. 10% FBS or Slit2 were loaded into right channel that was previously used for cell-loading, while FMLP was loaded into left channel that was previously used for cell-loading. The rectangular symbols represent mean value of cells' final positions. FMLP was shown to induce WBCs' migration while Slit2 was shown to inhibit WBCs' migration. **b** Cancer cells and WBCs show opposite migration under effect of FBS (10%) and FMLP (200 ng/mL). MDA-MB-231 cells and WBCs were mixed with 1:1 ratio. Fluorescence image shows the final positions of the cell mixture (t = 24 hours). Immunofluorescence staining was used to identify MDA-MB-231 (Vimentin, Green) and WBCs (CD45, red). The arrows indicate the migration directions. **c** Cell distribution after 24-hour assay. Majority of MDA-MB-231 cells (blue) were separated from WBCs (red). **d** Influence of FMLP on MDA-MB-231 cancer cells' migration. FMLP shows little impact on the migration of MDA-MB-231 cells.

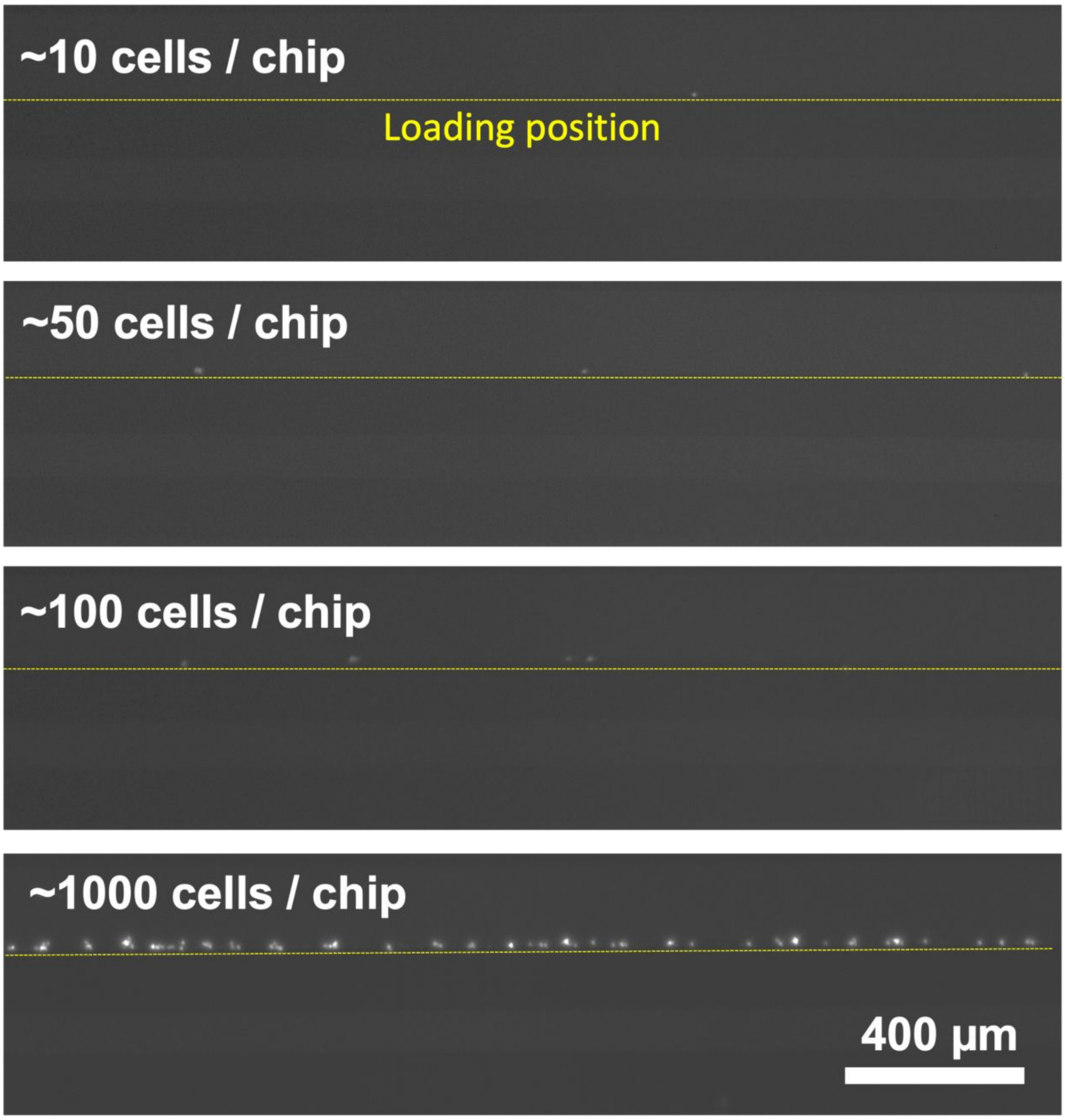

Figure S8. CTC-Race assay with a variable number of starting cells (~10, ~50, ~100, and ~1000 cells). These cells were MDA-MB-231 and their nuclei were stained with DAPI.

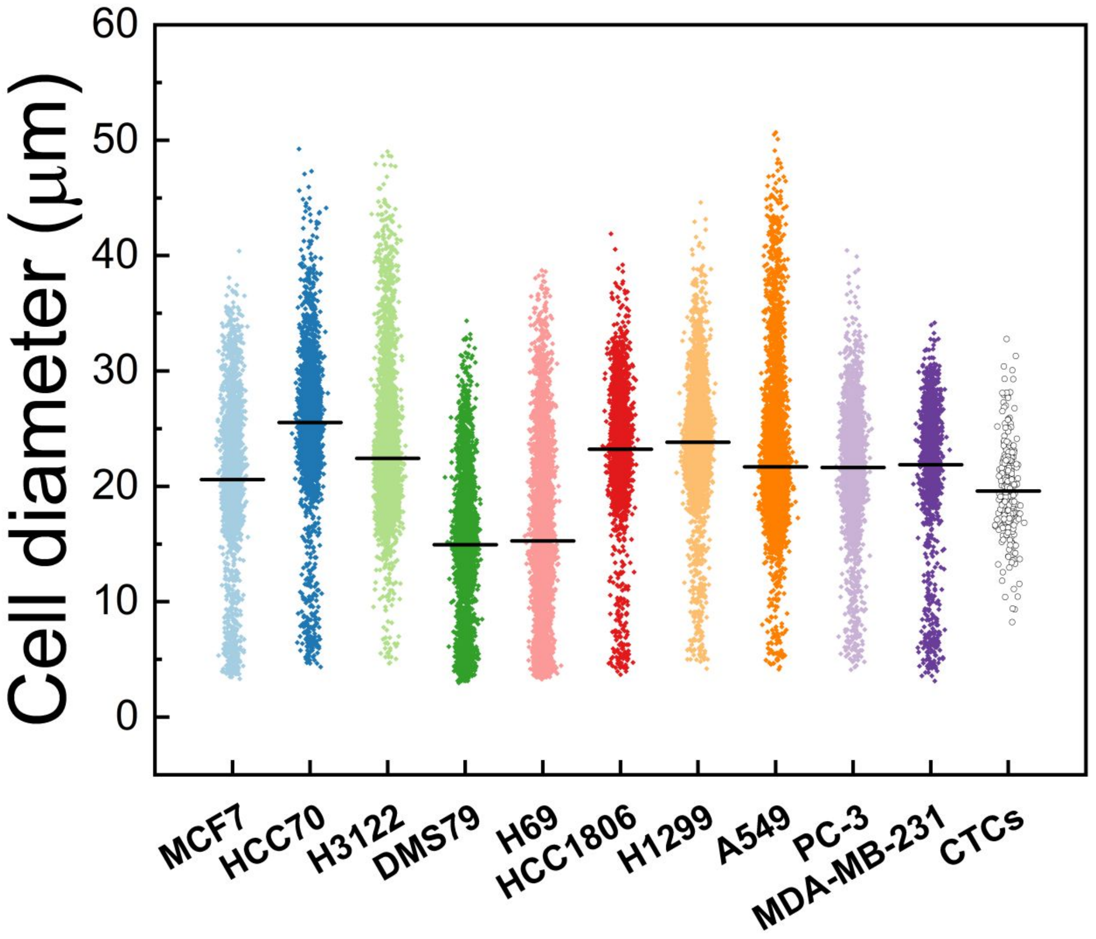

Figure S9. Diameter distribution of cultured cancer cell lines and patient-derived CTCs. The cell diameters(mean ± s.d.) for MCF7, HCC70, H3122, DMS79, H69, HCC1806, H1299, A549, PC-3, MDA-MB-231, and CTCs are 20.6 ± 6.7 μm (n = 2551), 25.5 ± 5.7 μm (n = 3343), 22.4 ± 5.1 μm (n = 6011), 14.9 ± 5.6 μm (n = 3310), 15.3 ±7.4 μm (n = 4195), 23.2 ± 5.6 μm (n = 2178), 23.8 ± 4.1 μm (n = 6045), 21.7 ± 5.2 μm (n = 7573), 21.6 ± 4.6 μm (n = 3329), 21.9 ± 6.2 μm (n = 1437), and 19.6 ± 4.0 μm (n = 266).

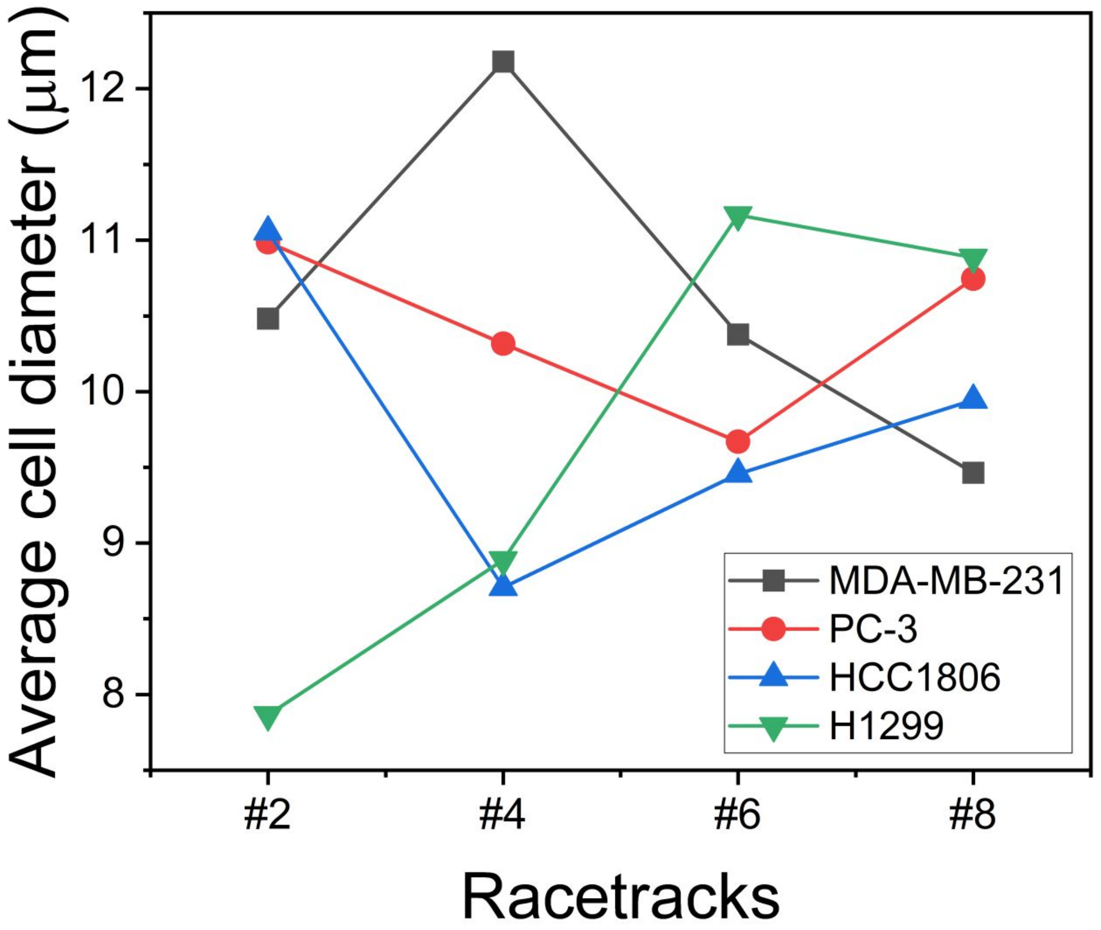

Figure S10. Relationship between mean cell diameter and mean cell migration distance for cultured cancer cell lines. The increasing numbers of Racetracks in the x-axis indicate increasing migration distance as explained in main text Figure 2.

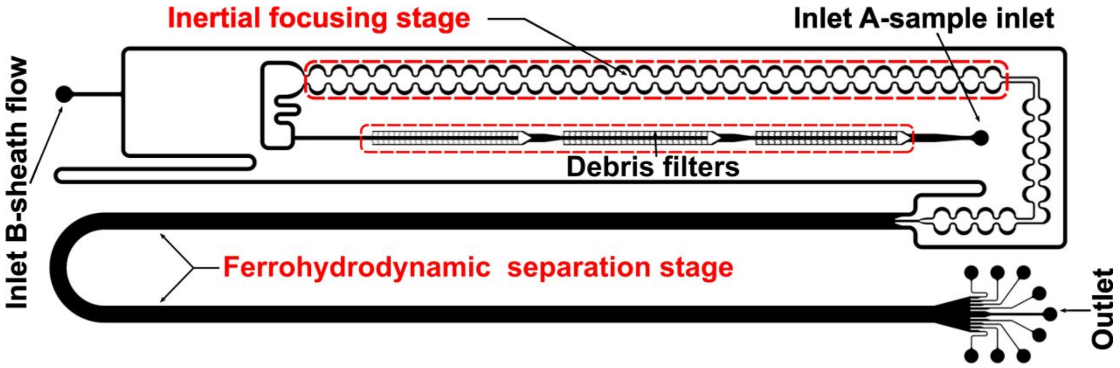

Figure S11. Schematic of the inertial-FCS cell sorting device. The debris filter stage of the device effectively removed debris and potential CTC clusters from the patient blood samples. The debris filter stage is designed to remove anything larger than 50 μm.

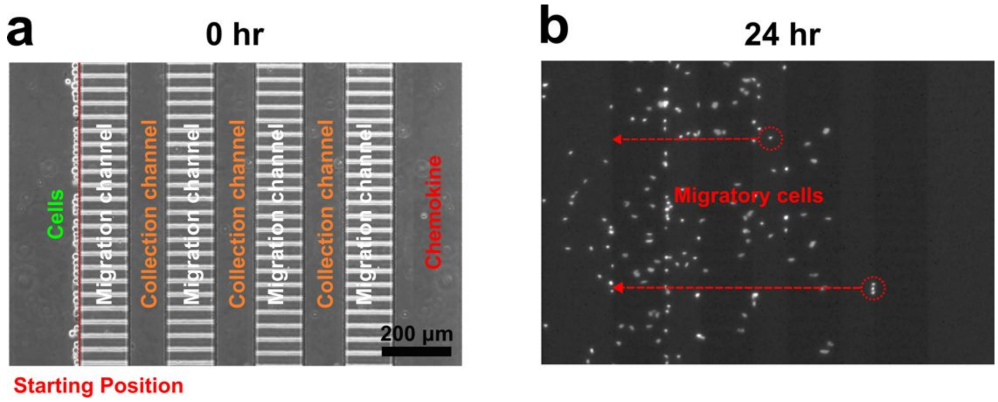

Figure S12. Protocol of tracking single cells in the migration channels of the CTC-Race device and the method of calculating migration distance and speed of cells. (a) CTC-Race device channels. At the beginning of the assay, all cells start their migration from the starting positions (red dashed line) indicated in (a). (b) At the end of the assay, migration distance and speed of cells are calculated assuming a straight-line migration trajectory. Using the locations of cells (nucleus of cells in the circles of (b)) within the device, we could calculate the total migration distance of the cells to be the straight-line difference between starting points and nucleus of cells (red dashed lines in (b)). The migration speed of the cells is defined as the total migration distance divided by the total assay time.

Table S1. Patient information and CTC isolated from each patient

	Age	Gender	Cancer type/stage	Blood volume processed for CTCs (mL)	Number of CTC per 1 mL of blood identified	Blood volume for IF staining (mL)	Blood volume of CTC-Race assay (mL)
**Patient 1**	75	Male	Squamous Cell Carcinoma of the lung, IV	10	252	5	5
**Patient 2**	69	Female	NSC adenocarcinoma, IIIB	8	72	4	4
**Patient 3**	79	Female	NSC adenocarcinoma, IV	20	173	5	5
**Patient 4**	-	Male	NSCLC favoring Squamous Cell, IV	15	221	5	5

Table S2. Purity of motile CTCs from patient samples in the CTC-Race assay

	Seeded CTCs	Remaining CTCs after assay	Migrating CTCs in the assay	Migrating WBCs	Purity of migrating CTCs
**Patient 1**	1260	207	186	5	97.31%
**Patient 2**	288	53	39	1	97.43%
**Patient 3**	865	167	82	1	98.78%
**Patient 4**	1105	159	93	2	97.89%

Table S3. Additional clinical information of patients

	Cancer invasiveness	Biopsy location	Genomic analysis type
**Patient 1**	Metastatic	Right endobronchial mass	Lung tissue/Foundation Medicine
**Patient 2**	metastatic to contralateral lung	Left lingula BAL	Blood/tissue
**Patient 3**	metastatic to brain	left hilar mass FNA	lymph node/Tempus
**Patient 4**	metastatic to brain	lung lymph node	lymph node/Tempus

Table S4. STR profile of cancer cell lines used in this study

	**MCF7**	**MDA-MB-231**	**HCC1806**	**HCC70**	**A549**	**H1299**	**H3122**	**DMS79**	**H69**	**PC-3**
**Catalog Number**	HTB-22	HTB-26	CRL-2335	CRL-2315	CCL-185	CRL-5803	Discontinued	CRL-2049	HTB-119	CRL-1435
**STR Profile**	D3S1358: 16	D3S1358: 16	Amelogenin: X	Amelogenin: X	D3S1358: 16	Amelogenin: X		Amelogenin: X,Y	D3S1358: 16	Amelogenin: X
	TH01: 6	TH01: 7,9.3	CSF1PO: 12	CSF1PO: 10,14	TH01: 8,9.3	CSF1PO: 12		CSF1PO: 10	TH01: 8,9	CSF1PO: 11
	D21S11: 30	D21S11: 30,33.2	D13S317: 11	D13S317: 12	D21S11: 29	D13S317: 12		D13S317: 11	D21S11: 30,31.2	D13S317: 11
	D18S51: 14	D18S51: 11,16	D16S539: 10	D16S539: 9,13	D18S51: 14,17	D16S539: 12,13		D16S539: 12	D18S51: 12,15	D16S539: 11
	Penta_E: 7,12	Penta_E: 11	D5S818: 13	D5S818: 12,13	Penta_E: 7,11	D5S818: 11		D5S818: 10	Penta_E: 12	D5S818: 13
	D5S818: 11,12	D5S818: 12	D7S820: 10,12	D7S820: 10,11	D5S818: 11	D7S820: 10		D7S820: 9,11	D5S818: 11,13	D7S820: 8,11
	D13S317: 11	D13S317: 13	TH01: 8	TH01: 9	D13S317: 11	TH01: 6,9.3		TH01: 8	D13S317: 12	TH01: 6,7
	D7S820: 8,9	D7S820: 8,9	TPOX: 8,9	TPOX: 10	D7S820: 8,11	TPOX: 8		TPOX: 8	D7S820: 9	TPOX: 8,9
	D16S539: 11,12	D16S539: 12	vWA: 16,18	vWA: 13,15	D16S539: 11,12	vWA: 16,17,18		vWA: 18	D16S539: 11	vWA: 17
	CSF1PO: 10	CSF1PO: 12,13	D3S1358: 16	D3S1358: 16,17	CSF1PO: 10,12	D3S1358: 17		D3S1358: 18	CSF1PO: 10,12,13	D3S1358: 16
	Penta_D: 12	Penta_D: 11,14	D21S11: 29	D21S11: 30	Penta_D: 9	D21S11: 32.2		D21S11: 30	Penta_D: 9,11	D21S11: 29,31.2
	Amelogenin: X	Amelogenin: X	D18S51: 16	D18S51: 13,16	Amelogenin: X,Y	D18S51: 16		D18S51: 14,17	Amelogenin: X,Y	D18S51: 14,15
	vWA: 14,15	vWA: 15,18	Penta_E: 12	Penta_E: 9,16	D8S1179: 13,14	Penta_E: 11		Penta_E: 7	vWA: 16,17	Penta_E: 10,17
	D8S1179: 10,14	D8S1179: 13	Penta_D: 15	Penta_D: 10	TPOX: 8,11	Penta_D: 13		Penta_D: 11,13	D8S1179: 13	Penta_D: 9
	TPOX: 9,12	TPOX: 8,9	D8S1179: 14,15	D8S1179: 13	FGA: 23	D8S1179: 10,13		D8S1179: 12,14	TPOX: 10	D8S1179: 13
	FGA: 23,25	FGA: 22,23	FGA: 25	FGA: 19.2,24	D19S433: 13	FGA: 20		FGA: 21	FGA: 24	FGA: 24
	D19S433: 13,14	D19S433: 11,14	D19S433: 14,15	D19S433: 12.5,15.2	D2S1338: 24	D19S433: 14		D19S433: 13.2,15	D19S433: 15,15.2	D19S433: 14
	D2S1338: 21,23	D2S1338: 20,21	D2S1338: 17	D2S1338: 25	vWA: 14	D2S1338: 23,24		D2S1338: 17,25	D2S1338: 17	D2S1338: 18,20
